# Physiologically based kinetic (PBK) modelling and human biomonitoring data for mixture risk assessment

**DOI:** 10.1016/j.envint.2020.105978

**Published:** 2020-10

**Authors:** Julia Pletz, Samantha Blakeman, Alicia Paini, Nikolaos Parissis, Andrew Worth, Anna-Maria Andersson, Hanne Frederiksen, Amrit K. Sakhi, Cathrine Thomsen, Stephanie K. Bopp

**Affiliations:** a European Commission, Joint Research Centre (JRC), Ispra, Italy; b School of Pharmacy and Biomolecular Sciences, Liverpool John Moores University, Byrom Street, Liverpool L3 3AF, UK^2^; c Oceansea Conservación del Medio Ambiente, Cádiz, Spain^2^; d Department of Growth and Reproduction, Rigshospitalet, University of Copenhagen, Copenhagen 2100, Denmark; e Norwegian Institute of Public Health, Oslo, Norway

**Keywords:** Physiologically based kinetic modelling, Human biomonitoring, Chemical mixtures, Biomonitoring equivalents, Human biomonitoring guidance values

## Abstract

Human biomonitoring (HBM) data can provide insight into co-exposure patterns resulting from exposure to multiple chemicals from various sources and over time. Therefore, such data are particularly valuable for assessing potential risks from combined exposure to multiple chemicals.

One way to interpret HBM data is establishing safe levels in blood or urine, called Biomonitoring Equivalents (BE) or HBM health based guidance values (HBM-HBGV). These can be derived by converting established external reference values, such as tolerable daily intake (TDI) values. HBM-HBGV or BE values are so far agreed only for a very limited number of chemicals. These values can be established using physiologically based kinetic (PBK) modelling, usually requiring substance specific models and the collection of many input parameters which are often not available or difficult to find in the literature.

The aim of this study was to investigate the suitability and limitations of generic PBK models in deriving BE values for several compounds with a view to facilitating the use of HBM data in the assessment of chemical mixtures at a screening level. The focus was on testing the methodology with two generic models, the IndusChemFate tool and High-Throughput Toxicokinetics package, for two different classes of compounds, phenols and phthalates. HBM data on Danish children and on Norwegian mothers and children were used to evaluate the quality of the predictions and to illustrate, by means of a case study, the overall approach of applying PBK models to chemical classes with HBM data in the context of chemical mixture risk assessment.

Application of PBK models provides a better understanding and interpretation of HBM data. However, the study shows that establishing safety threshold levels in urine is a difficult and complex task. The approach might be more straightforward for more persistent chemicals that are analysed as parent compounds in blood but high uncertainties have to be considered around simulated metabolite concentrations in urine. Refining the models may reduce these uncertainties and improve predictions. Based on the experience gained with this study, the performance of the models for other chemicals could be investigated, to improve the accuracy of the simulations.

## Highlights

• Human Biomonitoring (HBM) provides valuable insight into co-exposure to multiple chemicals.
• HBM data can be interpreted in comparison to biomonitoring equivalents of health based guidance values.
• Two generic physiologically based kinetic models were tested for deriving biomonitoring equivalents.
• Uncertainties and limitations were identified and discussed.
• The use of biomonitoring equivalents in assessing chemical mixtures was illustrated in a case study.

## 1 Introduction

Human biomonitoring (HBM) allows assessment of exposure to chemicals by measuring these compounds, their metabolites or other biomarkers of exposure present in body fluids (blood, urine, saliva, breast milk) or other biological matrices (hair, nails and teeth). In addition, monitoring of indoor and outdoor media (air and dust), and chemical exposure associated with food intake via diet diaries can be collected together with information on study participants' age, sex, socioeconomic background to give a more complete picture of external and internal exposure and to link human exposure to exposure sources and epidemiological survey data.

HBM programmes have been developed for decades. One example is the American HBM programme (National Health and Nutrition Examination Survey, NHANES), the largest ongoing project running since 1971 (McDowell, 1971). In Europe, various projects have been carried out over the years (Choi et al., 2015). In the Democophes project, mother–child pairs over large parts of Europe were studied (Den Hond et al., 2015) and in 2017 a project was launched by the European Commission called “The European Human Biomonitoring Initiative” (HBM4EU) which is a joint effort of 30 European countries. The aim of the project is to collect and interpret HBM data throughout Europe in order to safely manage chemicals and protect human health (HBM4EU, 2019). The long-term goal is to build bridges between the research and policy worlds and deliver benefits to society in terms of enhanced chemical safety.

Interpretation of HBM data is particularly valuable in the context of assessing combined exposure to multiple chemicals. The environment we live in is made up of an assortment of chemicals, some of which are potentially harmful if ingested in certain concentrations or combined with other chemicals. HBM data provide a measure of internal co-exposure to multiple chemicals and aggregate exposure to single chemicals across routes and sources of exposure.

The best way to interpret chemical concentration data is to compare them to a concentration that is deemed safe. Currently, the majority of these safe values are established as an intake dose, known as an acceptable or tolerable daily intake (ADI or TDI) or reference dose (RfD), referring to how much of one chemical a person can ingest daily over a lifetime without diminishing their health (FAO and WHO, 2009). Only recently have studies begun to look into establishing safe levels in urine or blood against which measured values can be compared. These are known as Biomonitoring Equivalents (BE) or HBM health based guidance values (HBM HBGV) (Angerer et al., 2011). Such reference values can serve as first screening values to evaluate potential risks based on chemical concentrations measured in biological samples such as human blood or urine, for individual chemicals and eventually also for combinations of multiple chemicals.

**Fig. 1:**
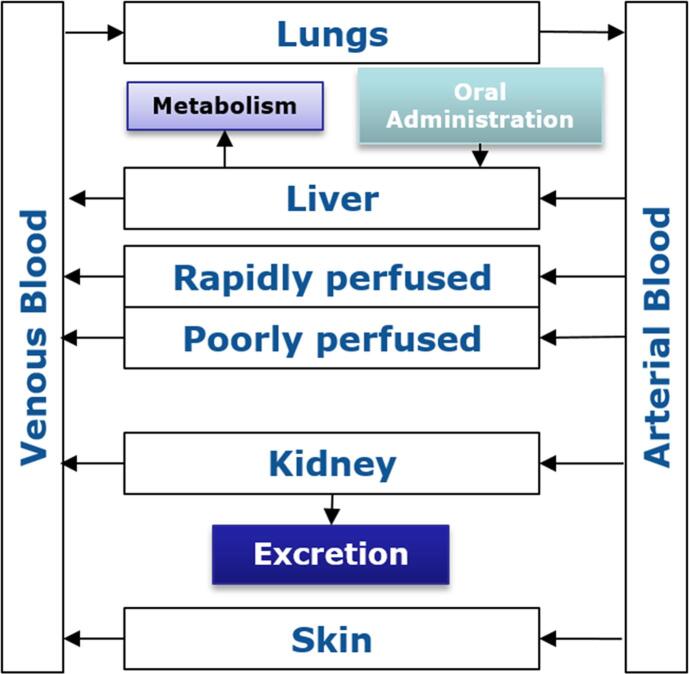
Schematic representation of a PBK model.

Some of the existing BE values have been related to biomonitoring data with the use of physiologically based kinetic (PBK)^3^ models (WHO, 2015), such as the one shown in Fig. 1. These models are based on mathematical descriptions of physiological characteristics (tissue volumes, blood flow, etc.) and biochemical processes (V_max_, K_m_, etc.) (Krishnan et al., 1994). PBK models describe the body as a set of interconnected compartments, which represent the organs and blood, describing the absorption, distribution, metabolism, and excretion (ADME) properties of a chemical or drug within the body (Krishnan and Andersen, 2007). For a given exposure scenario, PBK models predict the time-course of a parent chemical, its metabolite(s) or biomarkers of exposure as target tissue concentrations in an organism. PBK models can be used in two ways, either via forward dosimetry or reverse dosimetry (Hays et al., 2007). The former uses an intake dose and estimates the concentration of a compound in human fluids, whilst the latter uses the measured concentration in a body fluid and identifies the dose the person was exposed to. Either way, results from PBK models can be used to refine the setting of safe levels. In addition, PBK models can also be used to compare subgroups (sex, age, and sensitive population), characterise inter-individual variation, perform a range of extrapolations (e.g. acute to chronic exposure, route to route, low to high doses), and support read-across between substances by comparing kinetic profiles of structurally similar substances.

PBK models can be chemical specific or generic. A chemical specific PBK model is built using knowledge of the physicochemical / ADME properties and Mode of Action (MoA) for the specific chemical under study. For instance, distribution to relevant target organs and specific metabolite formation should be measured and included (Kawai et al., 1994, Abbiati and Manca, 2017, Hoffman and Hanneman, 2017, Moss et al., 2017). On the other hand, a more generic PBK model has a defined compartmental structure for all chemicals tested. These models are usually built on IT platforms (see Madden et al., 2019 for an overview of such platforms). This allows computing an entire chemical list with the same type of input data and the same assumptions and limitations. Furthermore the simulation of more than one chemical can be carried out at once and hence simplifies the task of carrying out a mixture risk assessment (Desalegn et al., 2018).

In a recent survey on the use of HBM data in chemical risk assessments, the lack of HBM guidance values was identified as an obstacle (Louro et al., 2019). The objective of our investigation was therefore to examine the suitability of using generic PBK models for deriving BE values based on TDIs or RfDs that can then be used for mixture risk assessment using HBM data at a screening level. The suitability of generic PBK models was investigated since it is very resource intensive to build specific PBK models for each single chemical and to derive their input parameters. In addition, the application of generic PBK models made it possible to compare multiple chemicals in a more homogenous way.

Several exposure platforms and models are currently available for analysis of HBM data, e.g. Stochastic Human Exposure and Dose Simulation (SHEDS) model, ConsEXPO, EUSES, Integra, IndusChemFate, HBM Simulator, MerlinExpo, and R/Httk. The aim of this study was to use generic PBK model platforms, which were chosen based on the following criteria: easy to use, readily available, full documentation and manual of use, transparent and simple, and easy to manipulate in case minor amendments were needed. For this purpose, five of these platform/models (Integra, IndusChemFate, HBM Simulator, MerlinExpo, R/Httk) were screened to match with inclusion exclusion criteria (supplementary material section S2 and Table S2). Based on this analysis and the criteria listed above, two generic tools were considered in this project, the IndusChemFate (ICF) tool and the High-Throughput Toxicokinetics (Httk) package, which are further described in Section 2.2. The correct mathematical implementation of the model structures were also verified by examining available details of the differential equations used (and/or the availability of the mathematical model code) and the parameterisations of the PBK model. The maintenance of mass-balance as well as blood flow balances within the model should be supported; equations and parameter values should be devoid of syntax or mathematical errors. Furthermore, it should be ensured that there are no numerical errors (World Health Organization, 2010). To this end the PBK model platforms were evaluated using the criteria laydown by WHO (2010) and by OECD (Sachana, 2019, OECD, 2020) and results reported in Supplementary Material S4.

**Fig. 2:**
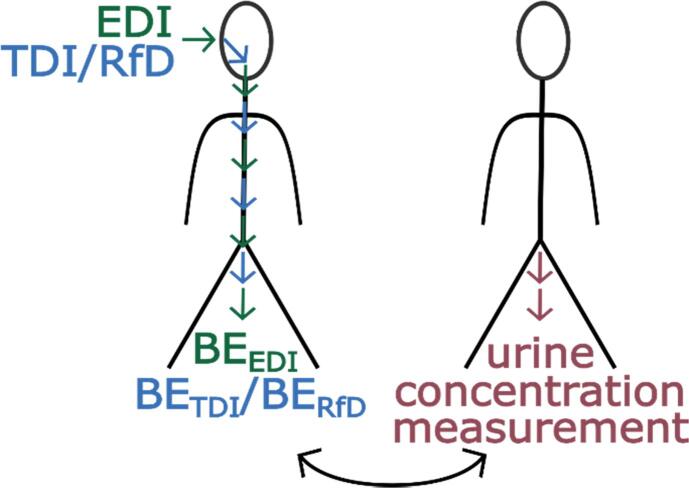
Use of Tolerable Daily Intake (TDI), Reference Dose (RfD) and Estimated Daily Intake (EDI) values as input doses in a forward dosimetry approach to obtain BE_TDI_, BE_RfD_ and BE_EDI_ values for risk assessment. The derived BE values can subsequently be compared to actual measured urinary concentrations.

Both models were used in a forward-dosimetry approach with established TDI or RfD values to obtain urinary BE concentrations, i.e. BE_TDI_ and BE_RfD_. Subsequently, with the information collected, a mixture risk assessment was carried out using HBM data obtained in Norwegian and Danish cohort studies (see Fig. 2). The mixture risk assessment was done for illustration purposes showing how such data might potentially be applied. This case study should not be regarded as a detailed risk assessment, but as a proof of concept.

## 2 Methodology

**Fig. 3:**
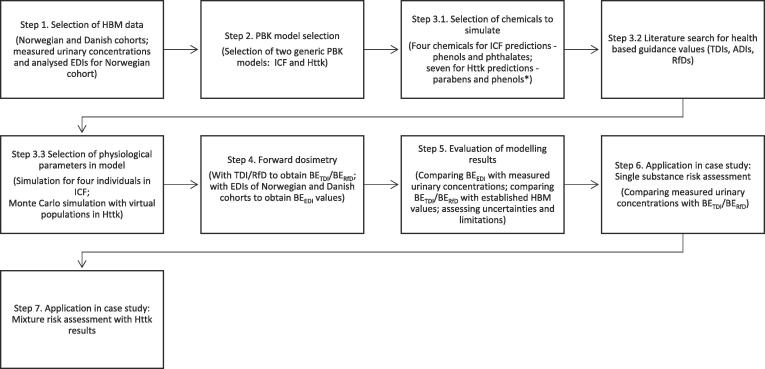
Workflow for the application of ICF/excel and the Httk/r, highlighting the steps used for the HBM analysis. Only Httk results were used for mixture risk assessment (MRA). A limitation was that the metabolism could not be taken into account in the current version of the Httk/r; the version 1.8, (2018). *Chemicals included in the analysis are: Phenols: Bisphenol A, Triclosan, Benzophenone-3; Phthalates: Di-n-butyl phthalate (DnBP), Butylbenzyl phthalate (BBzP); Parabens: Methyl paraben, Ethyl paraben, n-Propyl paraben, n-Butyl paraben. HBM: Human biomonitoring, TDI: Tolerable daily intake, EDI: Estimated daily intake, BE: Biomonitoring equivalent.

The overall approach is represented in Fig. 3, which reports the steps taken for the HBM analysis with the choice of chemicals (parabens, phenols, phthalates), the application of a Monte Carlo sampling approach for generation of ‘virtual populations’ (to capture the response for a representative population), and the steps for the illustrative risk assessment.

### 2.1 Step 1: Selection of human biomonitoring data

In order to identify HBM studies covering the analysis of many different chemicals and chemical groups per subject, we searched the literature and the HBM module of the Information Platform for Chemical Monitoring (IPCHEM, https://ipchem.jrc.ec.europa.eu). Among the relevant data sets identified, two were available and kindly provided under Data Transfer Agreements. The HBM data used in this study were from a Norwegian and two Danish cohort population obtained between January and May 2012, and between 2006 and 2007, respectively. In the respective studies, several classes of chemicals have been analysed, but for the study presented here, we focused on phthalates and phenols as substance groups. For the Norwegian study, we used the urinary concentrations and daily intakes estimated from measurements in external sources such as food, air, and dust.

Evaluation of urinary output is most straightforward if urine collection is complete (i.e., all output over periods of 24 h or more is captured). In practice, this can be very difficult, especially in some population groups such as children. Consequently, spot samples are often gathered instead. Spot samples are single void urine samples, which can be collected at any time during the day. If a single spot sample is to be collected per day, first morning voids (FMVs) are often selected on the basis of the assumption that the FMV provides an integrated sample over a relatively long part of the day and is therefore more likely to be representative (Kissel et al 2005).

Changes in the volume of fluid excreted will result in variability of urinary parent compound and metabolite concentrations irrespective of exposure to the chemical (Pearson et al., 2009). This is usually dealt with by standardising the measured crude concentration of the compound of interest against the urine dilution expressed as either specific gravity, osmolality or creatinine. Urinary creatinine can be used to correct for urinary concentrations because its production and excretion are relatively constant due to homeostatic control mechanisms, and creatinine excretion is mostly independent of urine flow (Boeniger, Lowry, & Rosenberg, 1993). We have resolved this by using standardised urinary concentrations in the Norwegian (specific gravity adjusted). For the Danish data sets we used unadjusted urinary concentrations. Lassen et al (2013) investigated the variability for repeated measurements of BPA and seven other phenols in spot, morning and 24 h urine samples. Normalisation of concentrations to osmolality, volume and creatinine did not substantially change the intraclass correlation coefficients (ICCs) for the repeated measurements.

The Norwegian dataset is based on a mother–child study cohort (48 mothers aged 32–56 years and 48 children aged 6–11 years). Urine samples were taken from 48 mothers and 46 children as morning spot samples (Cequier et al., 2017). Concentrations of several chemicals were measured from each of these urine samples; 23 adult females and 28 children have missing values for single chemicals because of interferences or below limit of detection (LoD). Further details about the enrolment, dietary intake (recording of food consumption), sample collection, and analysis have been published elsewhere (Cequier et al., 2015, Cequier et al., 2017). For some compounds, also food, dust, and air samples were analysed to determine external exposure from which estimated daily intakes (EDIs) were calculated (Cequier et al., 2014, Sakhi et al., 2014, Liagkouridis et al., 2017).

Specific gravity (SG) was measured in all samples and spanned from 1.003 to 1.032 (mean = 1.015) in mothers and from 1.009 to 1.032 (mean = 1.024) in children. The urinary concentrations were calculated according to the equation proposed by Boeniger et al. (1993) and using the SG values of the participants in the calculation (Cequier et al., 2017, Cequier et al., 2015).

One of the major limitations in working with short half-life biomarkers is the within individual variability. In the Norwegian study, the 48 mothers collected 2–8 urine samples over a period of 24 h, and the children collected 2–3 samples (at least morning and afternoon), (Cequier et al., 2015). 44% of the mother participants collected all spot urine samples, 27% participants collected more than 3 spot urine samples and 29% collected 2–3 spot urine samples (Sakhi et al., 2017, Sakhi et al., 2018). The calculated 24-h ICCs (as published in their study) were moderate to high for most of the phthalates and phenols indicating that the within individual variation is less compared to between individual variation during 24-h (Table S1.1 and S1.2).

The Danish population was made up of subsets of two cohort studies: firstly data from spot urine samples of 849 children aged 4–10 years from the Copenhagen Mother-Child Cohort (http://www.edmarc.net/mother-child-cohort.html; Boas et al., 2010, Frederiksen et al., 2014), secondly from 24 h urine samples of 129 children and adolescents between 6 and 21 years of age from the Copenhagen Puberty Study collected in 2007 (http://www.edmarc.net/puberty-cohort.html; Frederiksen et al., 2011, Frederiksen et al., 2013, Frederiksen et al., 2014). For the purpose of our study, only urine measurements in children aged 6–11 years were considered in order to compare the Danish group of children to the same age group of Norwegian children. This sub-group consists of 725 children in total (660 from the Copenhagen Mother Child cohort and 65 from the Copenhagen Puberty Study). Phthalate metabolites were measured in both Danish cohorts, while phenols such as BPA, TCS and BP-3 were measured in the Copenhagen Mother-Child Cohort and parabens were measured in the Copenhagen Puberty Study. For the Danish data sets we compared non-normalised and creatinine-adjusted concentration measurements (Table S1.3). The variability and range of concentrations did not change substantially after normalisation. Since the creatinine adjusted concentrations cannot be directly compared to the model outputs we used non-adjusted concentrations.

### 2.2 Step 2: Generic physiologically based kinetic model selection

A first search was conducted to find PBK model platforms of interest. This search resulted in a choice between 5 different platforms: Integra, IndusChemFate, HBM Simulator, MerlinExpo, and R/Httk. A table (supplementary material) was designed to compare the models and platforms. The desired platforms should be easy to use, readily available, with full documentation and manual of use, transparent and simple, and easy to manipulate in case minor amendments were needed. These considerations led to the selection of the platforms ICF and Httk for this study (details presented in Supplementary Material S2).

#### 2.2.1 The IndusChemFate tool

ICF was developed by IndusTox Consult & Santoxar, funded by the Long Range Initiative (LRI) of CEFIC within the context of the HBM2 project and is freely available on the CEFIC-LRI website (Jongeneelen and ten Berge, 2011, Jongeneelen and ten Berge, 2011b). The model is built in an MS excel spreadsheet, the source code is written in Visual Basic and is not password protected. The model contains 11 body compartments (i.e. lung, heart, brain, skin, adipose, muscles, bone, bone marrow, stomach and intestines (lumped), liver, and kidney) and calculates parent compound and one or more metabolite concentrations in organs over time, as well as the amount excreted in urine. This allows for the study of phthalates as they are rapidly metabolised (Frederiksen et al., 2007) and the concentration of some of their metabolites are an adequate indicator of exposure to the parent compound (Ramesh Kumar and Sivaperumal, 2016). By default, the model assumes physiological and anatomical parameters of a 70 kg reference human; however, several different subjects (e.g. male or female adult or child, with normal weight or obese) can be selected.

#### 2.2.2 The High-Throughput Toxicokinetics (Httk) package

The High-Throughput Toxicokinetics (Httk) package can be found in the CRAN r project (https://cran.r-project.org/web/packages/httk/index.html) which was created by the U.S. EPA's National Center for Computational Toxicology and constitutes a compilation of a one, three, and seven-compartment PBK (physiologically based toxicokinetic) models intended to compute concentration vs. time curves. A series of additional in-built functions calculate steady state concentrations, the number of days to reach steady state, and uncertainty and variability using a Monte Carlo analysis. Oral - via ingestion - was the exposure route selected to make predictions and the seven compartmental model was applied. All data used to parametrise the Httk model (physiological, tissue, as well as physicochemical data sources) are described in Pearce et al., 2017, Wambaugh et al., 2018. In contrast to ICF, the current version of the Httk model has no option to simulate metabolism and consequently predict parent substance and metabolite concentrations - only the concentration of the parent substance is predicted following metabolism, which is why the model could not be used for phthalates. The Httk/r version used in the current study was 1.8, (2018), the following functions were used: httkpop_generate, get_httk_params, “solve_pbtk”, “calc_css”.

#### 2.2.3 Sensitivity analysis on model platforms

Sensitivity analysis can broadly be described as a systematic investigation that leads to an understanding of how changes in the model input parameters can influence the simulation outputs. The one-at-a-time (OAT) sensitivity analysis was performed to understand to what extent the model output is affected by changing the value of an input parameter by 10% (increase/decrease). Sensitivity analysis should be performed for all parameters that are likely to markedly influence the outcome of the simulated kinetics. This includes key experimentally determined parameters (such as Ki, Vmax, Km) and parameters with a variety of values reported in the literature (such as kdeg).

For IndusChemFate the body and liver weight, the metabolism, Vmax and Km were changed by 10% to understand their impact on the model output. On the other hand, for R/httk the highest impact was from the clearance value.

### 2.3 Step 3: Selection of chemicals, their reference values, and physiological parameters for the models

#### 2.3.1 Step 3.1 and 3.2: Selection of chemicals and their reference values

To perform the analysis, nine compounds were selected as these were measured in both populations (Table 1). Several other chemicals and metabolites were available in the cohorts data sets, but were not considered for this analysis and the mixture risk assessment (MRA) due to limitations of both PBK models. For ICF, a steady state was not reached for most chemicals and Httk was limited to compounds measured as parent compounds in urine as it does not include metabolism.

**Table 1:** Compounds selected for the HBM analysis and MRA, and their TDIs and RfDs, if available. For phthalates, in addition to the parent compound, information on the main metabolite measured in urine as an indicator of exposure is presented. (Abbreviations: Abb.-Abbreviation, NA – not available; –/– - not applicable; Reference: 1. EFSA, 2015; 2. U.S. EPA, 2019; 3. ITER, 2019; 4. Council of Europe, 2009; 5. Group TDI from EFSA OpenFoodTox, 2018; 6. Moos et al., 2017; 7. EFSA, 2005b; 8. EFSA, 2005a.)

Parent Compound	Abb.	CAS	TDIs/ADIs (mg/kg/day)	RfD (mg/kg/day)	Human urine metabolite measured	Abb.	CAS
Bisphenol A	BPA	80-05-7	0.004^1^	0.05^2^	–/–	–/–	–/–
Triclosan	TCS	3380-34-5	0.047^3^	0.3^2^	–/–	–/–	–/–
Benzophenone-3	BP-3	131-57-7	0.1^4^	NA	–/–	–/–	–/–
Methyl paraben	MeP	99-76-3	10^5^	NA	–/–	–/–	–/–
Ethyl paraben	EtP	120-47-8		NA	–/–	–/–	–/–
n-Propyl paraben	n-PrP	94-13-3	0.02^6^	NA	–/–	–/–	–/–
n-Butyl paraben	n-BuP	94-26-8	0.02^6^	NA	–/–	–/–	–/–
Di-n-butyl phthalate	DnBP	84-74-2	0.01^7^	0.1^2^	Mono-n-butyl phthalate	MnBP	131-70-4
Butylbenzyl phthalate	BBzP	85-68-7	0.5^8^	0.2^2^	Monobenzyl phthalate	MBzP	2528-16-7

Four chemicals were initially selected to test the ICF model. These chemicals were two phthalates (DnBP, BBzP) and two phenols (bisphenol A, triclosan). They were chosen based on the simplicity of their metabolism and the availability of the estimated daily intakes/tolerable daily intakes and measured urine concentrations.

For Httk, four of the compounds under consideration were parabens (i.e. methyl paraben, ethyl paraben, n-propyl paraben and n-butyl paraben) and two phenols (i.e. bisphenol A and triclosan). Furthermore, Benzophenone-3 was included in the Httk section to complete the phenol group and therefore improve the MRA. The tolerable daily intakes (TDIs) established by public authorities were collected from databases or the literature and are presented in Table 1 including the source used.

#### 2.3.2 Step 3.3: Selection of physiological parameters for PBK modelling

##### 2.3.2.1 IndusChemFate input parameters

For the present work, various population scenarios were selected, e.g. normal child in rest or obese woman doing light work. For more information on how model-inherent parameters were defined or generated, see Jongeneelen and ten Berge, 2011, Jongeneelen and ten Berge, 2011b. The duration of simulation was set for an acute oral exposure at 24 h and for long-term exposure to steady state.

The physicochemical input parameters needed for each compound and its metabolites include the molecular weight, density, vapour pressure, log(octanol:water) partition coefficient at blood pH 7.4 (and skin pH of 5.5) and water solubility. Also, the model requires estimates of the oral absorption rate, resorption in renal tubuli, enterohepatic removal as well as V_max_ and K_m_ values with respect to each metabolic step that is intended to be considered.

Values of these parameters were sourced between mid July and end of August 2018 from the U.S. EPA Chemistry Dashboard, the Agency for Toxic Substances & Disease Registry (ATSDR), ECHA, PubChem and the public scientific literature (Eigenberg et al., 1986, Anderson et al., 2001, Völkel et al., 2002, Teeguarden et al., 2005, Aylward et al., 2009, European Commission Scientific Committee on Consumer Safety (SCCS), 2009, Coughlin et al., 2012, Hanioka et al., 2012, Koch et al., 2012, Takahara et al., 2014, Yang et al., 2015, Ashrap et al., 2017). Properties of the BPA glucuronide used are those saved in the ICF model.

Based on Pelkonen et al., 1973, Hakooz et al., 2006, Barter et al., 2007, Zhang et al., 2015, a factor of 40 mg/g was adopted to scale V_max_ values from a value established *in vitro* to an *in vivo* hepatic drug clearance value. The absorption rate (k_a_) was calculated using Winiwarter et al. (1998), model 3b, to derive the logarithm of the effective permeability (P_eff_) and Peters (2008) to then calculate the k_a_. All parameter values used for ICF simulations are presented in the supplementary material S3.

To test the ICF model, two females and two children from the Norwegian population were selected; one of “normal” weight (Woman/ Child 1) and one “obese” (Woman/ Child 2). To categorise the body mass index (BMI) of a child information on the age and sex are needed, however, a BMI of 16 is a normal weight for a child under 11 regardless of sex and a BMI of 22 is overweight or obese (CDC, 2018). Given this information, the weights of all four individuals were input into the code (via Developer -> VisualBasic) for the corresponding normal/obese woman/child at rest.

##### 2.3.2.2 Httk input parameters

Httk has a library of relevant parameters built into the model covering many chemicals, thus being very user friendly and limiting the time needed to gather input parameters. All data used to parametrise the Httk model (physiological, tissue, as well as physicochemical data sources) are described in Pearce et al., 2017, Wambaugh et al., 2018.

**Sample size for general population Monte Carlo simulations and generation of virtual population-specific physiological parameters**

The urine-related BE_TDI_ values are intended to be applicable to a diverse population, therefore it is more accurate if individual variability is taken into account. This variability can be met in Httk with the creation of a virtual population. Using the function “httkpop_generate”, a virtual population with physiological data taken from the NHANES (Johnson et al., 2014) may be generated. The sex, age range, weight category, kidney function category, and ethnicity may be defined and together they address the inter-individual variability. The characteristics of a created virtual population may then be used to generate population-specific parameters to run the PBK model.

We used the Yamane formula to define a sample size based on a given population size (Yamane, 1967):

(1) $$n=\frac{N}{\left[1+N\left(e^{2}\right)\right]}$$

where n = sample size, N = population, e = error tolerance.

The Yamane formula was used to characterise European Union general population size, based on a 508 million inhabitants, a sample size of 400 is estimated. Samples of 400 and 4000 persons were feasible in Httk, a Yamane-formula-based 400-subject and a 4000-subject population were created to simulate the BE_TDI_.

As done for the adult population, two populations, one with a larger sample size than the other, were created for the children. The result is a sample size of 1.000 made up of 500 males and 500 females and a sample size of 10.000 made up of 5.000 males and 5.000 females; all between the ages of 6 and 11 years.

These populations constitute a random selection of individuals from all weight categories (underweight, normal, overweight, and obese), “non-hispanic white” and “other” ethnicities, with normal kidney function and from 32 to 56 years of age for female adults and 6–11 for children. For each individual in these populations and each chemical, the function “get_httk_params” was used to generate parameters to run the PBK model. When running this function, poor metabolisers were considered.

### 2.4 Step 4: Forward dosimetry

As illustrated in Fig. 2, the forward dosimetry approach was used to predict internal reference values (threshold concentrations in urine), that can be used for screening level risk assessment, here called BE_TDI_ or BE_RfD_ depending whether the basis for deriving them was a TDI or RfD value, respectively.

In addition, forward dosimetry was carried out using estimated daily intakes as input to predict corresponding urinary levels, which could be compared to measured urine concentrations for evaluating the quality of predictions of the two models.

#### 2.4.1 Forward dosimetry using the ICF tool

The estimated daily intakes (EDIs) provided in the Norwegian HBM project for the chosen individuals based on measured concentrations in food, air, and dust, as well as the tolerable daily intakes (TDIs) and reference doses (RfDs) of the chemicals were input into the model as oral bolus doses (in mg/kg BW) at hour 0. No EDIs were measured for triclosan.

The PBK model was run at 10,000 iterations per hour for 24 h. The time to reach steady state was assessed using the equation T_ss_ = 5*t_(1/2)_, with t_(1/2)_ being the elimination half-life. Of all compounds under consideration, triclosan takes the longest time to reach steady-state, namely 145 h (6.04 days) if considering the maximum human elimination half-life of 29 h (European Commission Scientific Committee on Consumer Safety (SCCS), 2009). Therefore, to predict BE values at steady state, all compounds were run for seven days to make sure that all reached steady state at the end of simulation time. However, steady state was only reached for BPA. As results for each BE_TDI_, BE_RfD_ and BE_EDI_ and individual scenario (Woman/Child 1 and 2), the 24-h and steady state C_max_ values were recorded.

ICF predicts the concentration of the metabolites, which is directly applicable for the phthalates, where relevant metabolites have been analysed in the urine samples. For TCS and BPA, the ICF model predicts the urinary concentrations of the glucuronidated metabolites (TCS-glu and BPA-glu) while the hydrolysed TCS and BPA forms were analysed in the urine samples. In order to match the results and compare predicted with measured concentrations, the resulting urine concentrations of TCS-glu and BPA-glu were converted from µmol/L to ng/mL based on the molecular weight of the parent compound. The parent compounds are also predicted but result in concentrations that are very low, not affecting the concentrations obtained via the conversion of glucuronidated forms.

#### 2.4.2 Forward dosimetry using the Httk package

##### 2.4.2.1 BE_TDI_ / BE_RfD_ calculations

To calculate urinary BE_TDI_ values the TDI was input as a daily dose. Subsequently, “solve_pbtk” was run for each individual in each population. When the model was run for the time to reach steady state as defined by the function “calc_css”, it was found that the amount excreted in the urine was still increasing at the end of simulation time. Therefore, simulation times were defined as shown in Table S5.1 to ensure that steady state is reached in the urine by the end of the simulation.

The PBK output table generated for each individual includes the amount of the chemicals in the renal tubules. This amount was interpreted as amount excreted in the urine. The maximum amount excreted per day was divided by the volume of urine expected to be excreted (1600 mL for adult females and 820 mL for children (Sakhi et al., 2018)) and expressed as ng/mL.

BE_TDI_ calculations were performed on two sets of 4000 and 400 women and two sets of 10,000 and 1000 children to evaluate whether the BE_TDI_ values would differ considerably based on the different populations. The mean values differed very little between the larger populations versus the smaller ones but the standard errors of the mean of the larger populations were smaller and therefore the 4000 and 10,000 individual sized populations were used. When choosing between the two 10,000 and two 4000 individual populations the 5th percentile and median BE_TDI_ values were compared. The differences were marginal and the one population per age group with the overall lower BE_TDI_ values was chosen.

Of the distribution of 4000 urine TDI equivalents excreted per day in the adult female population and 10,000 values in the child population, the 5th percentile, as a conservative measure, and the median were used as BE_TDI_ values and compared with measured urine concentrations.

##### 2.4.2.2 BE_EDI_ calculations

For the evaluation of the model in Step 5, the calculation of BE values based on estimated daily intakes (BE_EDI_s) was used. The simulated BE_EDI_s were then compared to measured urinary concentrations. In this study we used two types of EDIs as input to the PBK models.

For the Norwegian Dataset, the EDIs were calculated based on measurements of potential exposure sources found in the households, such as concentrations in dust, air, and food (Sakhi et al. 2014). EDIs were provided in the Norwegian dataset for phthalates and BPA for 40–46 mothers and children, depending on missing values for some biomarkers. In line with the chemicals selected for this study, the only EDI values from the Norwegian dataset were thus for BPA for the use in Httk. For the calculation to be as accurate as possible and also allow for the inter-individual differences the adult females were split into three weight categories based on their body mass index (BMI): normal, obese, and overweight. As reported by the WHO, adults are considered overweight if the BMI is equal or greater than 25, and obese if the BMI is equal or greater than 30 (WHO, 2019). Twenty-eight (28) normal, 12 overweight, and 6 obese women with a mean weight of 62.2 kg, 75.2 kg, and 100.1 kg, respectively, were part of the Norwegian study.

Virtual populations were then created with the same number of individuals as each category. As a result the three virtual populations were: one of 28 normal weight individuals, one of 12 overweight, and one of 6 obese.

The children of the Norwegian study population have a mean weight of 34.3 kg. Their BMI values were presented. However, as their individual ages and sex are unknown, they could not be classified into the normal, overweight or obese categories. A virtual population of children (both male and female) between 6 and 11 years old and of all weight categories was created to see the ranges in weight and BMI. The results showed that, in Httk, any child with a BMI over 25 is considered obese, however, with BMIs under 25 the overlap between normal and overweight was too close to compare without the information of age. Therefore, looking at the HBM data of the children with EDIs and corresponding urine measurements, only one child is obese and the other 43 are normal/overweight. For the BE_EDI_ calculation of the obese child, an obese virtual population (200 boys and 200 girls) was simulated and the individual with the closest BMI and weight (weight_adj in Httk) to the child was chosen. For the other individuals, a virtual population of 200 boys and 200 girls with the weight categories normal and overweight was created. Individuals with weights or BMIs outside the limits of those measured in the population were then eliminated. To solve for the same number of individuals as in the data, 43 individuals were randomly selected until the maximum, minimum, median, and mean were similar to that of the HBM data.

To calculate the BE_EDI_ values for both adults and children, every individual's EDI was used as a daily intake for the individual's corresponding virtual population. Therefore, the same EDI has several BE_EDI_ values, one for each individual in the virtual population (e.g. 28 for the adult female normal weight population). For each EDI the average of its resulting BE_EDI_ values was then compared against the corresponding measured urine concentration.

To illustrate this with an example, e.g. a normal weight woman is selected having an EDI of 1.40E-04 mg/kg. This value is used as input for the normal woman virtual population of 28 individuals (n = 28), thus generating 28 BE_EDI_ values. The average BE_EDI_ (∑BE_EDI_/28) was 0.131 ng/mL. The measured urine concentration of the individual woman (with EDI = 1.40E-04 mg/kg) was 1.88 ng/mL, thus an order of magnitude higher than the simulated BE_EDI_ of 0.131 ng/mL.

**Fig. 4:**
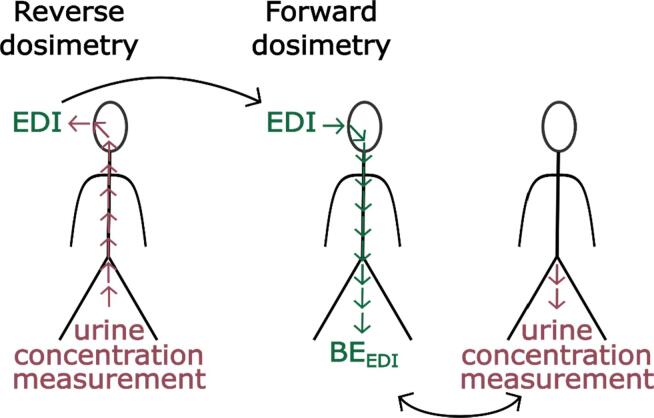
Calculation of EDI values from urinary concentrations in a reverse dosimetry approach and using these as input doses in a forward dosimetry approach to obtain BE_EDI_ values to evaluate model performance.

For the Danish study population, EDIs were not based on analytical measurements in exposure sources. Instead, EDIs were calculated in a reverse dosimetry approach in Frederiksen et al. (2013). We used 50th and 95th percentile EDIs for BPA and TCS (Table S5.2) to derive BE_EDI_ values using the virtual population of Danish children created for the calculation of the BE_TDI_ values (Fig. 4). Since the EDIs were derived from aggregate data as the 50th and 95th percentile EDIs for boys and girls between the ages of 6 and 10, separately, the virtual population was filtered for 6 to 10 year olds and split into boys and girls (4081 and 4092, respectively). Then the maximum, minimum, and median of the resulting BE_EDI_ values were compared against the 50th and 95th percentile measured urine concentrations.

### 2.5 Step 5: Evaluation of modelling results

To test the model and estimate the quality and validity of the BE_TDI_, all predicted values – i.e. BE_EDI_, BE_TDI_, BE_RfD_ – were contrasted with comparable values. BE_EDI_, which represent the predicted urine concentration reached at oral exposure to the EDI, were compared to the urine concentration measured in individuals. Under the assumption that EDI values are reliable, the closer BE_EDI_ are to measured urine concentrations, the better the model is considered to perform. BE_TDI_ and BE_RfD_, which constitute safe on-going urine concentrations, were compared to previously established BE values found in the literature or public databases.

### 2.6 Step 6: Illustrative case study: single substance risk assessment

The BE_TDI_ and BE_RfD_ values derived using the ICF and Httk models were compared to measured urinary levels. To interpret levels of internal exposure, the magnitude of exceedance of the established internal threshold and the percentage of the study population exceeding these threshold values were calculated. The risk quotient (RQ_ij_), Eq. (2), for each individual (i) is calculated for each chemical (j) by dividing the concentration of the chemical (j) found in the urine of an individual (i) by the BE_TDI_ values established in this report (see Table 3)

(2) $$RQ\left(ij\right)=\frac{\mathrm{Urine}\;concentration\;(ij)}{BE_{\mathrm{TDI}}\left(j\right)}$$

### 2.7 Step 7: Illustrative case study: Mixture risk assessment

To illustrate a possible risk assessment based on the combined exposure to multiple chemicals, a screening level mixture risk calculation was performed. Parabens and phenols are both known as endocrine disruptors, associated with effects on reproductive hormone and thyroid levels (Aker et al., 2016). In order to assess the risk that individuals face from these chemicals as a whole a Hazard Index (HI) approach was used. Ideally, only chemicals leading to the same adverse outcome should be considered for the combined assessment and in calculating the HI. However, including all chemical in a first screening level estimate independent of the detailed MoA and adverse outcome consideration is a worst-case conservative approach. If the resulting HI is smaller than 1, it indicates that there is low concern. If a potential concern is identified (i.e. if the HI > 1), a refinement looking more in depth into grouping chemicals that are expected to contribute to a specific effect should be performed, but this is out of scope of this case study.

The HI, Eq. (3), is calculated by summing all of the risk quotients (RQ), Eq. (2), of an individual, each RQ is the concentration of a chemical (j) found in the urine of an individual (i) divided by the BE_TDI_ values established in this report (see Table 3).

(3) $$HI\left(ij\right)=\sum RQ\left(ij\right)$$

## 3 Results

### 3.1 Deriving BE_EDI_ and BE_TDI_ values with IndusChemFate and comparison to measured concentrations of four individuals

#### 3.1.1 Calculations for Norwegian women and children

Using the calculated EDI from external sources of the Norwegian data set, the corresponding urinary concentrations BE_EDI_ were simulated with ICF and compared to the measured urinary concentrations in the Norwegian HBM dataset. Two female adults and two children were randomly selected from the normal and obese groups, to test the ICF model. For data protection reasons, individual urine concentrations and EDI values are not presented, but the factor of deviation between measured and predicted values are shown (Tables S5.3–S5.5).

The results from the 24 h simulation show that overall measured urine concentrations and estimated EDI-based urine concentrations differ by three to five orders of magnitude (Tables S5.3 and S5.4). However, the C_max_ urine concentration predicted for BPA only differs by approximately one order of magnitude from the measured urine concentration. For DnBP and BBzB, estimated EDI-based urine concentrations (BE_EDI_) are always lower than measured urine concentrations. This could indicate that there are other external sources of these chemicals that were not covered in calculating the EDI, but it can also indicate that ICF is underpredicting. The BE_EDI_ of BPA for the normal weight adult female and the obese child are higher than measured concentrations in urine. Unfortunately, there are no measurements to estimate EDIs for triclosan so no BE_EDI_ was calculated for this compound.

When running all compounds for the period of seven days, only BPA reaches a steady-state concentration. The venous blood concentration of all other substances continues to steadily increase, even when the simulation is run for a period substantially longer than seven days (100–300 days). Therefore, steady state simulations are only presented for BPA (Table S5).

Interestingly the steady state BE_TDI_, BE_RfD_ and estimated BE_EDI_ values are very similar to the 24-h C_max_ concentrations for BPA. All measured urine concentrations are below the calculated BE_TDI_ and BE_RfD_ values. Also, measured urine concentrations and those estimated from the EDI differ by approximately one order of magnitude, which can be considered a reasonable result.

It is difficult to assess the uncertainty underlying these results. In conclusion, predictions for BPA are more accurate than predictions for other substances in ICF since steady state was reached only for BPA. This may be related to the fact that the BPA was a test chemical for the development of the ICF model and its data are already provided in ICF when the model is downloaded from the Cefic-LRI website.

#### 3.1.2 Calculations for Danish children in IndusChemFate

As ICF only reaches steady state for BPA, this was the only chemical used. To evaluate the ICF model, the EDI values calculated in Frederiksen et al (2013), were input to ICF to recalculate the urinary concentration. The 50th and 95th percentile EDIs were used. To further evaluate the ICF model, the average weights of the boys and girls were calculated and input as both a scenario of normal weight child and obese child to visualise the difference in the model output. The two scenarios differ only by the fractions used to calculate the tissue volumes.

The results of this comparison show that ICF overestimates the concentration of BPA found in the urine based on the EDIs by a factor of 4–8. The normal child parameters are a closer fit for the bodyweights introduced and the predictions are closer for the 50th percentile EDIs (Table 2).

**Table 2:** Urinary concentration estimates of BPA and TCS. 50th and 95th EDI values obtained by reverse dosimetry from urinary concentrations, were used as daily doses in forward dosimetry in order to evaluate the generic models. The ICF results were obtained for BPA only, as TCS did not reach steady state in the model. The ICF scenarios for normal and obese child were used, however using the same input weight, i.e. a body weight of 35.2 kg for the boy and 31.6 kg for the girl, respectively. For the Httk model, virtual populations as described in Section 2.4.2.2 were used for the simulation of BPA and TCS. Using the 50th and 95th percentile EDI as input to the model, the resulting minimum (min), maximum (max), and median urinary concentrations for the virtual populations are shown.

Boys								
		EDI	HTTK simulated results (ng/mL)			ICF simulated results, normal child (ng/mL)	ICF simulated results, obese child (ng/mL)	Measured urine (ng/mL)
	Percentile	mg/kg/day	min	max	median			
BPA	50th	8.0E-05	0.01	0.88	0.04	13.00	14.82	3.33
	95th	3.1E-04	0.02	3.12	0.15	51.05	58.21	7.75
TCS	50th	2.8E-05	0.0002	0.03	0.001	–	–	1.45
	95th	1.4E-02	0.09	16.76	0.48	–	–	186.18

Girls								

		EDI	HTTK simulated results (ng/mL)			ICF simulated results, normal child (ng/mL)	ICF simulated results, obese child (ng/mL)	Measured urine (ng/mL)
	Percentile	mg/kg/day	min	max	median			

BPA	50th	4.97E-05	0.00	0.65	0.02	8.11	9.24	2.20
	95th	3.16E-04	0.02	4.16	0.15	51.54	58.77	11.20
TCS	50th	1.88E-05	0.0001	0.02	0.001	–	–	0.80
	95th	1.23E-02	0.06	12.63	0.41	–	–	37.01

### 3.2 Derivation of BE_EDI_ and BE_TDI_ values with Httk and comparison to measured concentrations

#### 3.2.1 Derivation of BE_EDI_ values for BPA in Httk

**Fig. 5:**
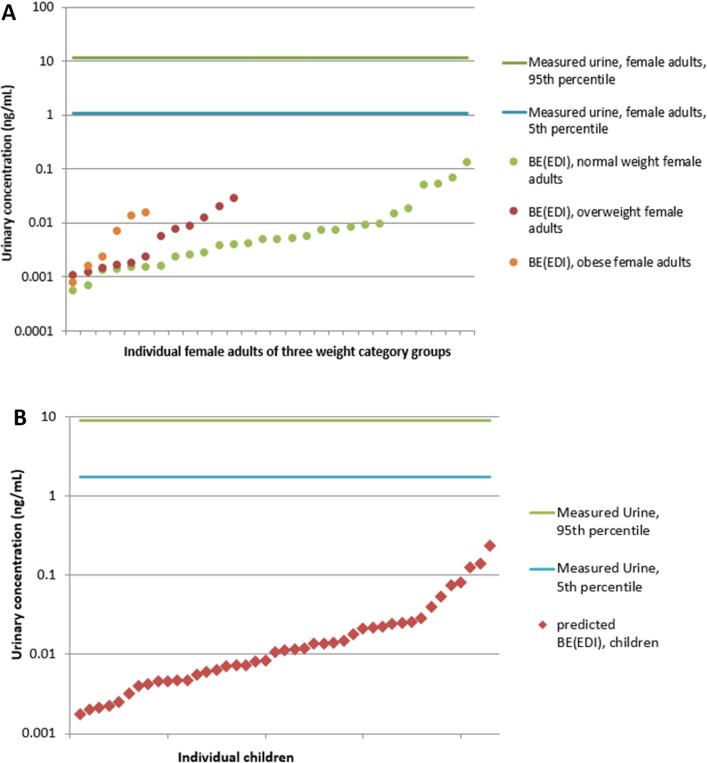
A. BE_EDI_ concentrations for BPA based on EDIs of 28 normal weight, 12 overweight and 6 obese female adults of the Norwegian group of women in relation to the 5th and 95th percentile of the measured urine concentration of this study population. B. BE_EDI_ concentrations for BPA based on EDIs of 43 Norwegian children in relation to the 5th and 95th percentile of the measured urine concentration of this study population.

In order to test the model and estimate the validity of forward dosimetry results in view of deriving BE_TDI_s, EDI values for BPA derived from ambient air, dust, and food concentrations for the Norwegian data set were used to calculate urine-based BE_EDI_ values which are subsequently compared to measured urine concentrations. Due to availability of EDI based on external exposure sources, this was only possible for BPA. Fig. 5A and B show the calculated BE_EDI_s for Norwegian women and children, respectively, compared to the range of measured urine concentrations, presented as 5th and 95th percentiles. The deviation between measured and simulated urinary concentrations is on average two to three orders of magnitude, with a minimum of one and maximum of five orders of magnitude. The higher the EDI input values used as daily doses, the closer the predicted results become to the measured urinary concentrations. This may indicate that either the higher the EDI, the more complete and reliable this value is, which results in a more reliable BE_EDI,_ or that Httk performs better with higher oral doses.

For the Danish data set, the BE_EDI_s were calculated using the EDIs estimated from reverse dosimetry, focusing on the 50th and 95th percentile EDIs. As shown in Table 2, for all cases (BPA and TCS, boys and girls, 50th and 95th percentile), the Httk model results underestimate the urinary concentrations. The predictions which are closest to the measured urinary concentrations are the maximum values which are underestimating by a factor of 2.5–3.8 for BPA and a factor of 3–43 for TCS. In all cases the results for the 95th percentile were better predicted than for the 50th percentile, as also seen for the Norwegian data set that Httk performed better at higher concentrations.

#### 3.2.2 Httk results for BE_TDI_ calculations

TDI values for the eight selected chemicals were used as input in Httk in order to calculate internal threshold BE_TDIs_. Virtual populations were run, consisting of 4000 female adults and 10 000 children which ingested daily doses of the TDI until steady state is reached in the urine. Table 3 shows the predicted BE_TDI_ values based on the 5th percentile and the median of the distribution of maximum urinary concentrations of virtual populations.

**Table 3:** BE_TDI_ values established in Httk in virtual populations of 4000 female adults and 10,000 children based on daily doses of the TDI.

	BE_TDI_ values (ng/mL)			
	Female adults, 5th percentile	Female adults, median	Children, 5th percentile	Children, median
**MeP**	1008.77	2971.53	716.06	1944.75
**EtP**	1886.90	5393.45	1361.21	3670.23
**MeP + EtP**	1217.50	4047.33	864.80	2693.16
**PrP**	4.73	13.92	3.41	9.45
**BuP**	4.56	12.86	3.19	8.65
**TCS**	0.89	2.57	0.64	1.73
**BP-3**	11.09	32.05	8.05	21.62
**BPA**	1.08	3.08	0.79	2.09

The 5th percentile BE_TDI_ values are all at least half the concentration of the median BE_TDI_ values. For MeP and EtP only a group TDI is established, therefore the BE_TDI_ is a result of the sum of MeP + EtP. The values for this sum were obtained by grouping all the urine TDI equivalents together and calculating the 5th percentile and median.

### 3.3 Comparison of derived BE_TDI_ values with previously established values

In order to better understand whether the BE_TDI_ values calculated in ICF and Httk are similar to previously established BE values of the compounds considered here, a search of comparable values was performed. Only TCS and BPA urine-based BE values were found in the literature. These had been established by the German HBM Commission using the term “HBM-I” values (Angerer et al., 2011) on the basis of TDI values published by Krishnan et al., 2010a, Krishnan et al., 2010b. These BE values are for TCS 3000 and 2000 ng/mL for adults and children, respectively, as well as for BPA 200 and 100 ng/mL.

Table 4 shows that for TCS, all BE_TDI_ values obtained from Httk and ICF differ from previously established levels by around four orders of magnitude. It needs to be taken into account, however, that for TCS the TDI value used for establishing the HBM-I-values was higher than the TDI used in our study. For BPA, however, existing BE values are in the same order of magnitude when considering C_max_-based BE_TDI_ values calculated in ICF, for female adults deviating only around a factor of 2.

**Table 4:** BE_TDI_ values calculated in ICF and Httk for female adults and children compared to previously proposed BE values (HBM-I-Werte) by the German HBM Commission.

		TCS	BPA	
**C_max_-based BE_TDI_ values established in ICF**	Normal weight female adult (ng/mL)	0.44^a^	427.92^b^	
	Obese female adult (ng/mL)	0.39 a	437.09^b^	
	Normal weight child (ng/mL)	0.73 a	649.20^b^	
	Obese child (ng/mL)	0.67 a	739.19^b^	

**BE_TDI_ values established in Httk**	Female adults, 5th percentile (ng/mL)	0.89	1.08	
	Female adults, median (ng/mL)	2.57	3.08	
	Children, 5th percentile (ng/mL)	0.64	0.79	
	Children, median (ng/mL)	1.73	2.09	

**Existing BE values (HBM-I-values)**	Adults (ng/mL)	3000^c^	2500^d^	200^e^
	Children (ng/mL)	2000^c^	1500^d^	100^e^

a BE_TDI_ values for TCS were derived in ICF using 24 h simulations, since a steady state was not reached.

b BE_TDI_ values for BPA were derived in ICF using steady state simulations, which is the best comparable to the concept of deriving TDIs as a safe dose based on chronic daily exposure. The difference between 24 h simulations and steady state simulations for BPA in ICF are however small (see Table S5.3–S5.5).

c HBM-I-values for TCS are based on a TDI of 0.12 mg/kg BW and day as opposed to 0.047 mg/kg BW and day which was used for BE_TDI_ values established in this study

d HBM-I-values for BPA based on a TDI of 50 µg/kg BW and day by EFSA (German HBM Commission 2012; Apel et al., 2017)

e HBM-I-values for BPA based on a TDI of 4 µg/kg BW and day by EFSA [2015] (update by German HBM Commission 2015; Apel et al., 2017)

The ICF-related values shown here are only based on two individual scenarios offered by the model. Therefore, a range of BE_TDI_ values reflecting inter-individual variability is not obtained with this model.

The BE_TDI_ values for TCS are much lower than the established HBM-I-values, thus being more conservative, which is also the case for Httk derived values for BPA. Only for ICF derived BE_TDI_ values are higher and therefore less conservative, even if they are closest to the previously established values.

### 3.4 Evaluation of ICF and Httk results: uncertainties and assumptions

Overall, it is difficult to evaluate the validity of the ICF and Httk results as the only exercise undertaken to test the model was the forward dosimetry approach using EDI values of both Norwegian populations. This exercise showed that simulated urine level BE_EDI_ values differ by on average two to three orders of magnitude from measured urine concentrations in 46 adult females and 43 children. These partly exceed the generally accepted difference of one order of magnitude between predicted and measured results. These results show that further improvements could be achieved by introducing refined parameter values (calibration) of the PBK model platforms, this was however beyond the study design.

It is crucial to consider that, besides uncertainties inherent in the ICF and Httk predictions, several uncertainties are likely to be associated with the EDI values used. Typically, these uncertainties relate to source variability (e.g. changes in emission rates), variability of model input parameters to calculate the EDI, and metabolic variability within a population and with respect to changes in metabolic rate of an individual which are not considered when calculating the intake (Hines et al., 2011). The approach used to calculate EDI values for Norwegian adult females includes assumptions of the amount of drinking water consumed and indoor air inhaled as well as three scenarios for dust ingestion in order to achieve sufficiently certain results (see Haug et al., 2011). Another aspect contributing to uncertainty of BE_EDI_ results is related to the use of the EDI value as a whole as oral dose even though a fraction of it is related to inhalation exposure. Furthermore, if other exposure sources, not captured via the monitoring of food, dust etc. are contributing substantially to the overall exposure, the internal exposure will of course also be underestimated (National Research Council, 2009, Bang et al., 2012, Yao et al., 2018). Relevant dermal uptake has been shown for semi-volatile chemicals, e.g. for phthalates from clothes (Morrison, Weschler and Bekö, 2017).

Interesting to note is that Httk predicted BE_EDI_ concentrations (in ng/mL) follow the same trend as EDIs (mg/kg BW) which indicates linearity between model input and output reflecting linear biological processes. However, metabolism may for instance saturate and therefore be non-linear.

ICF predictions for BPA are considered more accurate than predictions for other substances in ICF. The reason for this is primarily that steady state was only reached for BPA. Because of this, ICF results are not considered for single substance risk assessment (Step 5) and mixture risk assessment.

When compared to previously established TDI-based BE concentrations in urine, the BE_TDI_ values obtained with Httk appear to be conservative. Additionally, reference values such as TDIs and RfDs also bear uncertainties (e.g. extrapolation from animal to human populations, see also Hays et al., 2007) and are continuously updated which indicates to some degree lack of robustness overall.

A summary of the limitations and uncertainties of the model is given in Table 5. A full evaluation of the two PBK modelling platforms is available in the supplementary material S4.

**Table 5:** Assumptions, limitations and uncertainties of the ICF and Httk tools.

	IndusChemFate (ICF)	High-Throughput Toxicokinetics (Httk)
Problem formulation	How can PBK models be used to interpret HBM data in the context of assessing the risk of environmental pollutants?	
Assumptions	V_max_ and K_m_ were searched in the literature and scaled up to same unit to input into the model. Absorption rate calculated using a QSAR model. Predictions of input parameters based on QSAR. Intake is assumed to come from the oral route, although it is known that it can also come from air, dust or food. Urinary excretion is driven by the lipophilicity. The QSPR calculating solubility assumes that human blood consists to 0.7% of lipids. 8% of arterial blood is turned into primary urine. No tubular resorption of very soluble substances with a log(K_ow_) < −1.5. Physiological parameters do not change by sex or over time.	A simple PBK Model will represent the complexity of the human body. All Httk input parameters are correct, except the BW and the p value related to the intrinsic clearance of TCS which were changed (p = 0.06). Intake is assumed to come from the oral route, although it is known that it can also come from air, dust or food. Perfusion-limited kinetics. R_blood2plasma_ is constant throughout the body. Clearance is assumed to be relative to the amount unbound in whole blood instead of plasma, but converted to use with plasma concentration. Negligible blood volume fractions in all tissues to justify dividing by the tissue volume without a blood volume fraction and partition coefficient dependency in PBK tissue concentration equations. Wetmore data assumed f_ub_ = 0.005 for chemicals with f_ub_ below the limit of detection. Linear, non-saturated metabolism.
Input parameters	Sourced from different databases and the public literature which used a wide variety of techniques; Uncertainty in *in vitro* data (e.g. nominal as opposed to effective concentration is recorded); *In vitro* to *in vivo* extrapolation uncertainty (e.g. uncertainty related to the scaling factor used for V_max_ values); Only metabolism in liver was considered; Lack of detailed consideration of specific protein binding, interaction with intestinal flora, intestinal transport, and excretion by faeces. Prediction for individuals only; body weight can be amended; Lack of certain input parameter values (e.g. V_max_ and K_m_).	Fewer variety of sources and higher consistency of methodology; Uncertainty in *in vitro* data (e.g. nominal as opposed to effective concentration is recorded); *In vitro* to *in vivo* extrapolation uncertainty; High degree of QSAR-generated parameters; Allows only for metabolism in liver; Prediction for populations which are based on U.S. NHANES data; degree of variability to European/Scandinavian population unclear.
Model structure	High number of compartments; Steady state not reached for most compounds; Mass balance can easily be checked but shows errors, especially in children's populations;	Moderate number of compartments; Simulation of metabolite kinetics unavailable; Conversion from chemical amount in renal tubule to urine concentration necessary;
Model output	Uncertainties underlying EDIs and TDIs; Uncertainties in urine concentration measurements, in particular related to the use of a one spot measurement of non-persistent compounds.	Uncertainties underlying EDIs and TDIs; Uncertainties in urine concentration measurements; Size of adult female population above 4.000 individuals gave error message.

An OAT sensitivity analysis was performed for BPA using IndusChemFate showing that if there is a 10% increase/decrease of the value (Vmax and Km) this will influence the model output in urinary excretion. Also organ and body weight were changed by 10% but this did not influence the model output.

An OAT sensitivity analysis was performed for BPA using Httk showing that if there is a 10% increase/decrease of the value (CLint) this will influence the model output in urinary excretion.

### 3.5 Case study: applying derived BE_TDI_ values for single substance risk assessment

To interpret the measured urinary concentrations, they can be compared to toxicological and health based reference values. BE_TDI_ values can serve as such reference values to compare to. This study looked into possible generic methodologies and related limitations to derive such BE_TDI_ values. The application of the BE_TDI_ values derived here is performed for illustration of the possible use and cannot be considered as detailed risk assessment. Results need to be interpreted with care and uncertainties around the BE_TDI_ values need to be taken into account as discussed in Section 3.4.

The results based on the BE_TDI_ and BE_RfD_ values established using ICF are shown in Tables S5.3–S5.5. When considering the calculated BE_RfD_ and BE_TDI_ values in comparison to the measured urinary concentrations for the four chemicals, all four individuals appear to be exposed to concentrations below the BPA and Triclosan internal RfD or TDI values. For BBzP, urine concentrations measured in all four individuals are below the BE_TDI_ but for Woman 1 and Child 2 their measured urinary concentration is higher than that of the BE_RfD_. For DnBP, all measured urine concentrations are above the calculated BE values for all four individuals by one to two orders of magnitude. Urinary concentrations above BE_RfD_ or BE_TDI_ are indicating a potential risk.

**Fig. 6:**
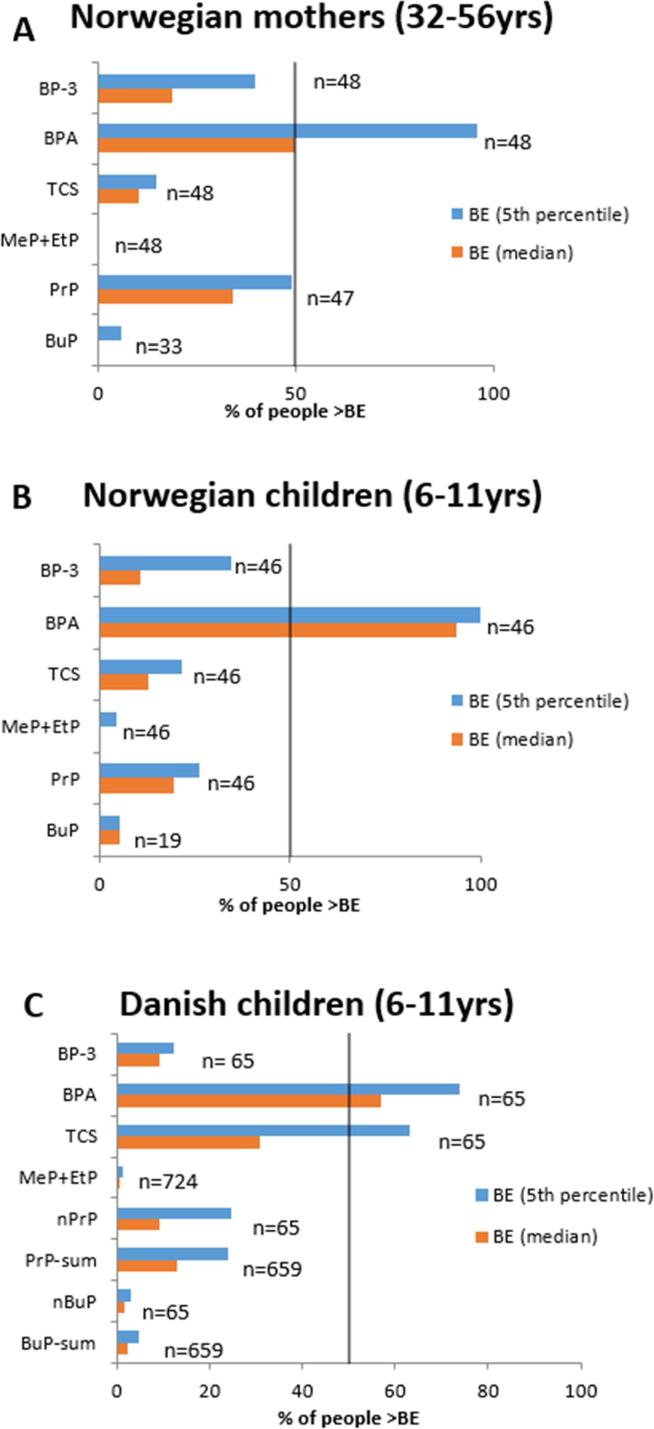
A. Number of individuals (in %) in the Norwegian adult females' group whose urine concentration exceed the 5th percentile and median based BE_TDI_ values. B. Number of individuals (in %) in the Norwegian children group whose urine concentration exceed the 5th percentile and median based BE_TDI_ values. C. Number of individuals (in %) in the Danish children group whose urine concentration exceed the 5th percentile and median based BE_TDI_ values.

Then, measured urinary concentrations were compared to the Httk simulated BE_TDI_ values, both 5th percentile and median (Fig. 6). Comparing the results for the different chemicals assessed, exposure to BPA seems to be of greatest concern with most exceedances of the derived threshold BE_TDI_s. Over all chemicals and populations, and considering both the 5th percentile and median based measures, 50% and over exceed the BE_TDI_ established for BPA. With respect to the 5th percentile based BE_TDI_, 95%, 100% and over 70% of Norwegian adult females, their children, and Danish children, respectively, exceed this level. While TCS exposure only exceeds both BE_TDI_ values in 10–21% of the Norwegian adult and child populations, 63 and 30% of the Danish children exceed the 5th percentile and median based measures, respectively. In both groups of children, both PrP BE_TDI_ values are exceeded by 9 to 26% of individuals while 34 and 48% of adult females show higher urine concentrations. Considering the 5th percentile based BE_TDI_ for BP-3, approximately 35 to 40 of both Norwegian populations show higher levels of exposure, whereas less than 20% (including the Danish group) exceed the median-based value. Exposures to MeP + EtP and BuP appear to constitute the least concern. Up to 6% of all population groups exceed both 5th percentile and median based BE_TDI_s for these chemicals.

As expected, the 5th percentile based BE_TDI_ may be a rather conservative measure. In the cases of BP-3 in Norwegian children and female adults, BPA in adult females, and n-PrP and TCS in Danish children, the difference in the number of individuals exceeding the 5th percentile and the median based BE_TDI_s is substantial. In cases where the BE_TDI_ is exceeded by roughly 20% or less of the population, the difference to the median-based BE_TDI_ does not seem to be significant.

### 3.6 Case study: Applying derived BE_TDI_ values for mixture risk assessment

As described above, the aim of this study was to investigate the suitability and limitations of using generic PBK models to derive BE_TDI_ values for a larger number of chemicals in a similar way. The idea is to facilitate the use of HBM data in risk assessment for a wider range of chemicals and in particular in the context of assessing mixtures. The application of the BE_TDI_ values derived here is performed for illustration of the possible use and cannot be considered as detailed risk assessment. Results need to be interpreted with care and uncertainties around the BE_TDI_ values need to be taken into account as discussed in Section 3.4.

In a screening level approach, the Hazard Index (HI) was calculated summing up the risk quotients for all chemicals independent of the specific endpoints of the chemicals. If the resulting HI > 1 it indicates a potential risk and the need for further refined assessments. The HI was calculated for each individual against the two BE_TDI_ values established using virtual populations in Httk, i.e. 5th percentile BE_TDI_ and median BE_TDI_. In addition, a HI was determined for each study population in order to represent the risk each one faces. These HIs were calculated using the median measured urine concentration of each chemical against the median BE_TDI_ calculated. It should be noted that the median is estimated for some chemicals with fewer samples than for others (i.e. n-BuP).

**Fig. 7:**
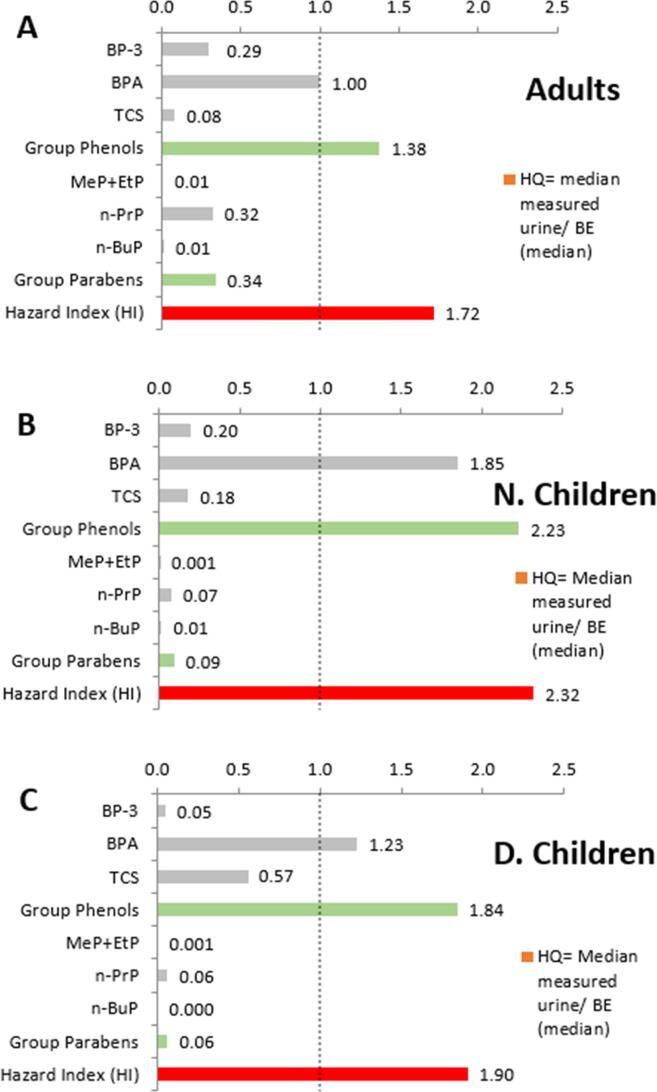
Comparison of median measured urine concentrations to threshold concentrations “median BE_TDI_^”^ established in this study using Httk. Risk Quotients for individual chemicals (grey bars), sum of risk quotients for chemical groups phenols and parabens (green bars), and the overall Hazard Index (HI, red bars) are presented for (A) Norwegian female adults, (B) Norwegian children, (C) Danish children. The risk quotients and HI using the BE_TDI_ values established in this study have to be interpreted with care, taking into account the uncertainties around the BE_TDI_ values and the orders of magnitude difference in them when using different models or comparing to formerly established BE thresholds. (For interpretation of the references to colour in this figure legend, the reader is referred to the web version of this article.)

The HI for all three study population groups (Norwegian female adults, Norwegian children and Danish children) is above the recommended maximum of 1 and is driven mainly by the phenols group (Fig. 7). More information is presented in the supplementary material (Figs. S5.1 and S5.2) investigating the impact of selecting different percentiles. In fact, the phenols make up over 95% of the HI for both children populations. In terms of chemicals, BPA has the highest HQ in all of the populations, whilst n-BuP and MeP + EtP have the lowest. In addition, two chemicals of note for the Norwegian mothers are BP-3 and n-PrP. This last one shows that the mothers are more exposed to parabens than their children, however, their HI is lower than both children populations. Finally, although Norwegian children have the highest HI they appear to be less exposed to TCS than the Danish children. In the comparison between the Norwegian and the Danish children, it needs to be taken into account however, that samples were taken in different years (2012 for Norwegian study and 2006/2007 for the Danish study), so that a decrease of external exposure concentrations over time might also play a role.

## 4 Discussion

The aim of this study was to investigate and illustrate the suitability of generic PBK models to derive biomonitoring equivalents of health based guidance values, such as BE_TDI_ values. The need for such values to facilitate the interpretation of HBM data has been acknowledged, however considering their limitations and using them as a screening tool (Louro et al., 2019; HBM4EU Deliverable D 5.4). Deriving internal reference values is a difficult task. When performed, all related uncertainties must be taken into consideration. Such values are established for example by the German HBM Commission for 19 substances using various data types and approaches. Furthermore, the EU project HBM4EU has started to derive HBM health based guidance values for their priority substances, also using PBK modelling. This study focussed on the applicability of generic PBK models, as these may assist in deriving such values for a wider range of chemicals that are measured in humans and are continuously growing in number.

ICF and r/Httk were used in this exercise, which are probably just one example of the mathematical models that could be used to interpret HBM data. However, to improve the overall HBM interpretation other examples of exposure platform like INTEGRA, MERLIN EXPO, and HBM Simulator could be investigated. These platforms are important tools for analysis of indirect exposure assessments allowing for estimation of individual or population exposure patterns.

### 4.1 Suitability of the two generic models ICF and Httk

Both, ICF and Httk, were selected among the publicly available PBK model tools that could be easily used for a wider range of chemicals. The case studies explored the suitability of applying a generic approach to deriving urinary reference values to facilitate the interpretation of HBM data in a chemical risk assessment context for single substances and mixtures.

ICF contains 11 body compartments and by default assumes physiological and anatomical parameters of a 70 kg reference human. However, several subjects can be selected, such as man, woman, and child, with normal weight or obese. The model contains algorithms as Quantitative Structure-Property Relationships (QSPRs) for blood:air, tissue:blood partitioning and for urinary excretion; estimating concentrations and amounts in body fluids (air excreted, urine and blood) and in organs after inhalation, oral intake or dermal exposure according to user defined exposure scenarios. For the present work, various population scenarios were selected, e.g. normal child in rest or obese woman doing light work. As organ and tissue volumes and blood flows are scaled relative to the body weight, these are amended for a prediction for a normal or obese woman or child. Besides calculating parent and metabolite concentrations in organs over time, the amount excreted in urine is also predicted. ICF was primarily selected because of its user-friendly qualities. Furthermore, conversion of a parent compound to one or more metabolites may be modelled in parallel. This is particularly relevant for phthalates as they are rapidly metabolised (Frederiksen, Skakkebæk and Andersson, 2007) and the concentration of some of their metabolites are an adequate indicator of exposure to the parent compound (Ramesh Kumar and Sivaperumal, 2016). A steady-state concentration was only reached for BPA whereas the venous blood concentration of all other substances continues to steadily increase, even when the simulation is performed for a period of 100–300 days.

Httk constitutes a compilation of a one, three, and seven-compartment PBK model intended to compute concentration vs. time curves. Here, the model was used for all urinary predictions, and the number of days to reach steady state was calculated for all compounds modelled. Models are parameterised with high-throughput *in vitro* data and structure-derived physicochemical properties of over 1300 compounds and physiological data from the most recent U.S. Centers for Disease Control and Prevention (CDC) National Health and Nutrition Examination Survey (NHANES) data. At present, there is no option to estimate metabolite concentrations in the current version of the Httk which is why the model could not be used for phthalates. The exposure route applied was oral - via ingestion and the seven compartmental model was used to run the simulations. Httk was primarily selected because of the richness of data made available within the model. Httk contains parameter data for n-propyl and n-butyl parabens but not their isomers. For certain individuals of the Danish dataset only measurements of the sum of n- and i-propyl as well as n- and i-butyl parabens are available. In these cases, data points based on sum measurements are plotted separately in the graph (see Fig. 7C).

One advantage of Httk is that the population variability can be considered by using virtual populations. Many different approaches exist to determine a sample size. For simulations considering the general population, Monte Carlo simulations with a sample size of 10,000 is often used (Slob, 2006, Sprandel et al., 2006, Goutelle et al., 2009, Punt et al., 2016). However, a study on the number of replications required in Monte Carlo simulations found that for all 22 studies considered, the minimum recommended number of replications (which corresponds to the sample size) is less than 10,000 (Mundform et al., 2011). Overall, 7500 to 8000 replications produce robust results, while in a number of cases 5000 may be enough (Mundform et al., 2011). Others use the Yamane formula to define a sample size based on a given population size (Yamane, 1967). In the present work we used the Yamane formula for the population of the European Union sized 508 million inhabitants, with estimation of a sample size of 400 people. Unfortunately, in Httk, a virtual population of 10,000 females could not be created and most attempts to create a population of 5000 females produced error messages. As samples of 400 and 4000 are feasible in Httk, a Yamane-formula-based 400-subject and a 4000-subject population were created to simulate the BE_TDI_ which were compared to urine concentrations measured in mothers. For the population of children, to be similar to the two mother samples, 500 males and 500 females of the same age group were produced. These populations constitute a random selection of individuals from all weight categories (underweight, normal, overweight and obese), “non-hispanic white” and “other” ethnicities, with normal kidney function and from 32 to 56 years of age for female adults and 6–11 for children. When the model was run for the time to reach steady state as calculated by “calc_css”, it was found that the amount excreted in the urine was still increasing at the end of simulation time. Therefore, simulation times were defined to ensure that steady state is reached in the urine by the end of the simulation.

The performance of the PBK models for the chemicals under investigation was evaluated in two ways: (1) for the Norwegian dataset, estimated daily intakes resulting from external exposure measurements in dust, air, and food were used to simulate related urinary concentrations, which were subsequently compared to measured urinary concentrations. (2) for the Danish dataset, a reverse dosimetry approach was used to calculate EDIs based on urinary concentrations. These EDIs were then used in the models to predict urinary concentrations and to compare to the measured urinary concentrations again. Higher predicted BE_EDI_ values can be considered more conservative. Predicted concentrations were mostly orders of magnitude lower than the measured urinary concentrations. This underprediction indicates that the BE_TDI_ values established in the same way tend to be too low, i.e. too conservative, indicating a risk at very low levels. This was also confirmed when comparing to formerly established HBM-I values by the German HBM Commission, which were much higher for TCS and BPA than the BE_TDI_s of this study.

The predictions of a generic PBK model may be more reliable if BE values are derived for a specific population by adapting the model’s physiological parameters, e.g. intrinsic clearance and kidney function, to said population. Another option for the future is to use chemical specific PBK models to establish general BE values for children and adults using more general assumptions about a wider (e.g. European) population to be used as a virtual population.

To further assess the suitability of the models, more compounds should be studied and the influence of selected parameters further investigated. The use of virtual populations and considering different percentiles offers one way of differentiating and taking into account of population variability. To further facilitate evaluation of model predictions, more data on external exposure would be of value in order to estimate more reliable EDI values.

The appropriateness and predictive ability of the two PBK models were further examined. We applied the WHO (2010) and OECD (Sachana et al., 2019) criteria to assess model credibility, although in the present study we use PBK model platforms and not compound-specific PBK models. As the WHO (2010) reports, there are potentially two types of uncertainties and built-in errors in the models and in the platforms:

Firstly, the structural uncertainty of the model was assessed (Supplementary Material S4). To this end, the PBK model platform equations and mass balance were checked. To our knowledge the biological basis was respected as was the mass balance (total absorbed mass was equal to the intake dose given). However, the total absorbed mass of the parent compound should also be equal to the sum of mass of parent + metabolites in all model compartments plus the mass excreted in urine and/or exhaled air (total sum). In the ICF model there is a 3% discrepancy which means the model underpredicts the “total sum”.

Secondly, the parameter uncertainty was evaluated. For ICF predictions, this information was searched in the literature and scaled according to an accepted IVIVE approach. It is unclear why steady-state concentrations were not reached for the majority of compounds tested. However, it may be worth searching for additional data (or deriving these via read-across) to increase knowledge of parameter ranges suitable for the populations of women and children.

In Httk, the steady state concentration (Css) predicted for most chemicals analysed by Wambaugh et al. (2018) was overestimated by more than an order of magnitude. Accordingly, the total clearance (CLtot) was underestimated, more for non-pharmaceuticals, which implies that urinary concentrations are likely to be underestimated. CLtot is derived from the sum of hepatic metabolism and passive glomerular filtration in the kidney (Pearce et al., 2017). Hepatic metabolism is calculated with a well-stirred model using scaled *in vitro* intrinsic hepatic clearance data and for both hepatic metabolism and glomerular filtration the fraction unbound is considered. Therefore, the chemical-specific parameters driving CLtot are hepatic clearance and the fraction unbound. For BPA, Wambaugh et al. (2018) showed that the *in vivo* estimated CLtot is approximately two orders of magnitude higher than the *in vitro* predicted CLtot. An underestimation of CLtot may be interpreted as either an underestimation of hepatic clearance, glomerular filtration in the kidneys or both. Metabolism of BPA is occurring rapidly, predominantly in the liver and via glucuronidation, and to a lesser extent via sulfation (Völkel et al., 2002, Kurebayashi et al., 2010). As mentioned earlier, measured BPA urine concentrations include hydrolysed BPA glucuronide (and sulfate) concentrations which are not included in Httk predicted urine concentrations of BPA. Besides the possibility that hepatic clearance and the fraction unbound of BPA may be underestimated which would explain the overestimation of Css, a major factor contributing to the difference between *in vitro* and *in vivo* CLtot is likely also related to a fast renal excretion of BPA glucuronide *in vivo* due to a high fraction unbound (estimated at 0.95 by Edginton and Ritter, 2009). No data was found in the literature and the Metrabase database (http://www-metrabase.ch.cam.ac.uk) indicating that BPA is excreted via processes other than glomerular filtration, such as active transport. Due to the underestimation of CLtot for BPA by two orders of magnitude, the results indicating that individuals’ exposed to BPA exceed the safe level need to be revisited, meaning that a more refined approach is needed to generate the BE_TDI_ for BPA.

Similar to BPA, TCS is found to be predominantly excreted as glucuronide in the urine of humans while concentrations of the parent and TCS sulfate are negligible (Arbuckle et al., 2015). As the calculated BE_TDI_ for TCS is related to the concentration of the parent only, comparing this value to the measured urine concentration, which is predominantly based on excreted TCS glucuronides, is suboptimal. A predicted BE_TDI_ accounting for both, TCS and TCS-glu, would be considerably higher.

However, since the aim of this study was to evaluate the performance of Httk without making vast amendments to inherent parameter values, the results were generated as presented above.

Additional assumptions and uncertainties for model and parameter uncertainties are reported in Table 5.

Finally comparing a model prediction with the HBM experimental data is a validation step that establishes confidence in the model. In the case of BPA using ICF the simulated urinary concentration was overestimated by a factor of 4 and 8, slightly higher than the two fold criterion of model acceptability by WHO (2010) but within the one order of magnitude, so still reasonable. To correct for this discrepancy in model overprediction the model could be refined or calibrated in order to better fit the values. However, we did not wish to refine the model structure or the input parameters, but rather to assess the “readiness” of such model platform as such. This is because the aim of the study was to investigate whether, based on the example of two generic PBK models, it would be feasible to derive BE_TDI_ values that could be used in screening level assessments of HBM data in a short time for a larger number of chemicals with different characteristics.

### 4.2 Limitations and strengths of comparing modelling results with urinary concentrations

One of the major limitations in working with short half-life biomarkers is the within individual variability. However, as described in Section 2.1, in the Norwegian study, the calculated 24-h intraclass correlation coefficients (ICCs) were moderate to high for most of the phthalates and phenols indicating that the within individual variation is less than the between individual variation during 24-h (Tables S1.1 and S1.2). Thus, we could use 24 h or steady state urine concentrations as modeling outputs for BETDI or BERfD and compare them with the HBM study. The 24-h ICCs of the studied phthalates (DnBP and BBzP) and phenols (BPA and TCS) are high. These moderate to high ICCs indicate that one spot urine sample can be used to estimate the exposure to phthalates and phenols.

Another important consideration in interpreting the data is the timing between exposure episode and urinary void. If exposure does not result in a steady state – which is often the case for many non-persistent chemicals as BPA - the concentration measured in spot (or morning) void will depend on the timespan since exposure occurred and the urinary void. While 24-h urine samples can even out variability in excretion over the day, there are also day to day variability which for BPA may be quite high even when comparing 24-h urine (Lassen et al., 2013). Also, the relevance of different routes of exposure and the aggregate exposure from them makes the calculation of estimated daily intakes more challenging (Søeborg et al., 2014). In interpreting the results of the comparison between model results and measured urinary concentrations, these uncertainties need to be taken into account.

### 4.3 Comparison of measured exposure concentrations to BE_TDI_ values for single substances and mixtures

Overall, the BE_TDI_ values established with the generic models are very conservative for TCS, i.e. much lower than the established HBM-I-values. Whereas for BPA, the values derived with ICF are less conservative than the HBM-I-values and the Httk derived values are again more conservative. It needs to be taken into account that also reference values established by various authorities might differ substantially, such as shown in Table 1 as deviations of one order of magnitude, between TDI and RfD values. Also reference values might be revised over time, such as observed for BPA during this study.

The results for single substances and mixture risk assessment have to be interpreted carefully considering the uncertainties discussed above. The case studies were performed for illustration purpose only. However, they suggest that the group of phenols (BPA, TCS) are of higher concern than the other chemicals that were assessed.

## 5 Conclusions

One strategy to facilitate the use of HBM data in risk assessment is focused on establishing safe levels in urine or blood against which measured HBM values can be compared. These safe levels are known as biomonitoring equivalents (BE) (Hays et al., 2007) or HBM health based guidance values (Angerer et al., 2011). Since HBM data provide information on realistic internal co-exposure scenarios for humans, they are of particular value for assessing risks from combined exposure. The availability of BE values will facilitate, at screening level, mixture risk assessments using HBM data. The aim of the study was therefore to investigate the use of generic PBK models for BE derivation and to illustrate the use of derived values for assessing the risks caused by exposure to multiple chemicals using HBM data.

This study applied two generic PBK models, ICF and Httk, to establish BE values, using HBM data from a Danish and a Norwegian cohort study. A limited number of compounds could be simulated, i.e. four chemicals in ICF and seven chemicals using the seven compartmental Httk model. These models were used to assess provisional biomonitoring equivalents (BEs), in a forward dosimetry manner, when applying reference doses (RfDs) or tolerable daily intakes (TDIs).

In the current study, ICF had the advantage of including metabolism features to address chemicals such as phthalates for which usually metabolite concentrations are analysed in urine samples. However, the model seemed to work only for BPA and was less reliable for other tested chemicals. Furthermore, it required a substantial number of input parameters which were not easy to find in the literature or simulate, in particular for the metabolites.

The use of Httk is an elegant solution as it has a library of relevant parameters built into the model covering many chemicals, thus being very user friendly and limiting the time needed to gather input parameters. However, in the current version (1.8, 2018), metabolism is only addressed via intrinsic clearance. This means that metabolite concentration predictions were not included, so that it can only be applied for chemicals where the parent is measured in the urine samples.

The study shows that establishing safety threshold levels in urine is a difficult and complex task. There are uncertainties in the simulated results of metabolite concentrations in urine as well as intraindividual and interindividual variances that must be taken into account, especially when based on spot urine samples. Refining the models may reduce these uncertainties and improve predictions. However, for certain chemicals, such as those that mainly remain as a parent compound, it may be more useful to obtain BE values in blood rather than urine.

The approach described herein is a promising complement to the current risk assessment procedure for single chemicals and mixtures. Based on the experience gained with this study, the performance of the models for other chemicals could be investigated. This will provide further insight into the uncertainties and their sources, and improve the accuracy of the simulations.

## Declaration of Competing Interest

The authors declare that they have no known competing financial interests or personal relationships that could have appeared to influence the work reported in this paper.

## Footnotes

3 The term PBK model is synonymous with physiologically based pharmacokinetic (PBPK), physiologically based biokinetic (PBBK), and physiologically based toxicokinetic (PBTK) models.
