# Supplementary material for: Physiologically based kinetic (PBK) modelling and human biomonitoring data for mixture risk assessment
Source: Environ Int. 2020 Oct;143:105978. doi: 10.1016/j.envint.2020.105978 (PMC7684529; doi:10.1016/j.envint.2020.105978)
Supplement: Supplementary data 1 [file mmc1.docx]

Supplementary Material

“Physiologically Based Kinetic (PBK) modelling and Human Biomonitoring Data for Mixture Risk Assessment”

**Authors**

Julia Pletz*^1,2^, Samantha Blakeman*^1,3^, Alicia Paini^1^, Nikolaos Parissis^1^, Andrew Worth^1^, Anna-Maria Andersson^4^, Hanne Frederiksen^4^, Amrit K. Sakhi^5^, CathrineThomsen^5^, Stephanie K. Bopp^1^

*These authors contributed equally

Affiliations

^1^ European Commission, Joint Research Centre (JRC), Ispra, Italy

^2^ present address: School of Pharmacy and Biomolecular Sciences, Liverpool John Moores University, Byrom Street, Liverpool L3 3AF, UK

^3^ present address: Oceansea Conservación del Medio Ambiente, Cádiz, Spain

^4^ Department of Growth and Reproduction, Rigshospitalet, University of Copenhagen, Copenhagen 2100, Denmark

^5^ Norwegian Institute of Public Health, Oslo, Norway

Contact person:

stephanie.bopp@ec.europa.eu

**S1 Limitations and Strengths of using spot urine or 24h urine samples**

**Table S1.1, ICCs of different phthalate metabolite concentrations (SG adjusted and ln transformed) during one day in Norwegian mothers (from Sakhi et al., 2017).**

| Phthalate metabolites | Within-person variance | Between-person variance | ICC (95% Confidence interval) |
| --- | --- | --- | --- |
| MEP | 0.40 | 1.76 | 0.81 (0.78-0.85) |
| MiBP | 0.21 | 0.20 | 0.49 (0.42-0.57) |
| MnBP | 0.24 | 0.59 | 0.71 (0.66-0.76) |
| MBzP | 0.26 | 0.56 | 0.68 (0.63-0.73) |
| MEHP | 0.34 | 0.68 | 0.67 (0.61-0.72) |
| MEHHP | 0.24 | 0.58 | 0.71 (0.65-0.75) |
| MEOHP | 0.25 | 0.63 | 0.72 (0.67-0.76) |
| MECPP | 0.13 | 0.48 | 0.79 (0.75-0.83) |
| oh-MiNP | 0.40 | 0.62 | 0.61 (0.54-0.67) |
| oxo-MiNP | 0.47 | 0.67 | 0.59 (0.52-0.65) |
| cx-MiNP | 0.19 | 0.52 | 0.73 (0.68-0.78) |

**Table S1.2, ICCs of environmental phenols (SG adjusted and ln transformed) in Norwegian mothers during one day (from Sakhi et al., 2018).**

| Environmental phenols | Within-person variance | Between-person variance | ICC (95% confidence interval) |
| --- | --- | --- | --- |
| MeP | 0.41 | 0.95 | 0.70 (0.65-0.75) |
| EtP | 0.46 | 2.82 | 0.86 (0.83-0.88) |
| PrP | 0.92 | 2.67 | 0.74 (0.70-0.79) |
| BPA | 0.20 | 0.46 | 0.70 (0.64-0.75) |
| BP-3 | 0.23 | 4.34 | 0.95 (0.94-0.96) |
| TCS | 0.12 | 3.75 | 0.97 (0.96-0.98) |

**Table S1.3, Percentiles of phenol, BP-3, and paraben measurements of Danish children and their creatinine adjusted concentrations**

| Concentrations | Percentiles | Copenhagen Puberty Study | | | | | | | Copenhagen Mother-Child Cohort | | | |
| --- | --- | --- | --- | --- | --- | --- | --- | --- | --- | --- | --- | --- |
|  |  | 24h urine samples | | | | | | | Spot urine samples | | | |
|  |  |  |  |  |  |  |  |  |  |  |  |  |
|  |  | BPA | TCS | BP-3 | MeP | EtP | n-PrP | n-BuP | MeP | EtP | PrP_sum | BuP_sum |
| [ng analyte/mL] | 10th | 0,07 | 0,25 | 0,00 | 1,80 | 0,00 | 0,00 | 0,00 | 1,05 | 0,00 | 0,00 | 0,00 |
|  | 25th | 0,75 | 0,44 | 0,41 | 3,45 | 0,00 | 0,00 | 0,00 | 3,07 | 0,00 | 0,00 | 0,00 |
|  | 50th | 2,57 | 0,98 | 1,05 | 7,30 | 0,81 | 1,12 | 0,00 | 8,12 | 0,51 | 0,55 | 0,00 |
|  | 75th | 4,90 | 2,08 | 2,37 | 17,05 | 1,40 | 3,26 | 0,00 | 23,22 | 1,67 | 3,19 | 0,30 |
|  | 90th | 7,57 | 46,58 | 19,07 | 55,82 | 5,08 | 10,63 | 0,76 | 92,31 | 6,22 | 14,02 | 1,29 |
| [µg analyte/ g creatinine] | 10th | 0,14 | 0,43 | 0,00 | 2,47 | 0,00 | 0,00 | 0,00 | 1,97 | 0,00 | 0,00 | 0,00 |
|  | 25th | 1,38 | 0,66 | 0,55 | 4,82 | 0,00 | 0,00 | 0,00 | 5,31 | 0,00 | 0,00 | 0,00 |
|  | 50th | 3,65 | 1,18 | 1,17 | 9,16 | 0,95 | 1,21 | 0,00 | 13,23 | 0,57 | 0,94 | 0,00 |
|  | 75th | 5,85 | 3,18 | 3,56 | 24,11 | 1,75 | 4,71 | 0,00 | 36,42 | 2,11 | 4,33 | 0,41 |
|  | 90th | 12,10 | 44,11 | 24,69 | 68,93 | 6,27 | 17,84 | 0,86 | 140,81 | 7,09 | 17,87 | 1,74 |

**S2 Comparison of available modelling platforms**

We screened for platforms in human and environmental health developed for toxicology and exposure assessment. The platforms should have the following criteria simple to use, simple to download, simple to implement, without commercial license, availability of model code to check the equation and if necessary to adapt it and reported platform validation. The following platforms (see Table S1) were selected due to their capability in prediction of HBM data. All these platforms have the possibility to compare predictions with biomonitoring data, which serve as a gold standard for validation of model predictions. Predictions of levels in target organs, allows for a comparison with measured data collected during biomonitoring campaigns; this provides a solid foundation against which of the models can be validated.

**Table S2:** PBK modelling platforms or exposure models, assessment and rational for including or excluding a platform from the analysis.

| Platforms  Element | Integra | IndusChemFate | HBM Simulator | MerlinExpo | R/httk |
| --- | --- | --- | --- | --- | --- |
| Links | <http://cefic-lri.org/toolbox/integra/> | <http://cefic-lri.org/toolbox/induschemfate/> | <http://cefic-lri.org/toolbox/hbm-simulator/> | <https://merlin-expo.eu/> | <https://cran.r-project.org/web/packages/httk/index.html> |
| Without commercial license | YES | YES | YES | YES | YES |
| Description | The INTEGRA platform is a tool to support a refined aggregate exposure assessment and human biomonitoring data assimilation. | IndusChemFate can be used to estimate the level of a chemical in various body tissues and body fluids following exposure. | HBM Simulator was developed to allow investigation of the representativeness of spot biomonitoring samples in relation to different exposure patterns, chemical-related properties including half-life, and estimate how this is affected. | MERLIN-Expo tool contains a set of models for simulating the fate of chemicals in the main environmental systems and in the human body. | High-Throughput Toxicokinetics developed to interpret human exposure to chemicals and in vitro to in vivo extrapolation |
| Format platform/model language | acslX | Excel – visual basic | MATLAB | Ecolego | R |
| Chemical Libraries | YES 150 substances | Yes 15 substances | four different metals (As, Cd, Mn, Ni), 15 organic compounds (incl. 9 parabens, triclosan, triclocaban, chlorophenone-1, -3, -8, and Bisphenol A) | YES 30 substances | YES of 987 substances |
| Simple to download and implement | YES, Registration needed | YES, excel format | Not So straight forward, Download a package | YES | Not so straightforward Knowledge of R or R studio needed need to download the package. |
| Simple to use | NO | YES | NO | NO | NO |
| Availability of model code | NO | YES imbedded  code is written in Visual Basic and is not password protected | NO  (behind interface) | NO | YES imbedded |
| Availability of equations , published literature | YES  Sarigiannis, et al.. 2020  doi.org/10.1016/j.envres.2020.109307. | YES (Jongeneelen & ten Berge, 2011 & 2012)  doi: 10.1093/annhyg/mer075.  doi: 10.1007/s00420-011-0713-9 | Not found references that are mentioned in the webpage and executive summary.  As off 30/03/2020 | YES several manuals <https://merlin-expo.eu/learn/documentation/model-documentation/> following CEN 2015 | YES Reference Manual <https://cran.r-project.org/web/packages/httk/httk.pdf> |
| MODEL UNCERTAINTY, VARIABILITY | Whereas parameter variability has been extensively addressed in the distribution functions, the aspect of model uncertainty is less addressed. In none of the tools, it is currently possible to distinguish between variability and uncertainty. In order to run a probabilistic assessment in INTEGRA, model users should describe distribution functions For the INTEGRA model, it is so far unclear to what extent certain (generic) model parameters distribution functions have been pre-filled in the models |  | Not reported | Whereas parameter variability has been extensively addressed in the distribution functions, the aspect of model uncertainty is less addressed. In none of the tools, it is currently possible to distinguish between variability and uncertainty. In order to run a probabilistic assessment in MERLIN-Expo model users should describe distribution functions of input parameters. For MERLIN-Expo, this is clearly described in the model documentation. |  |
| MODEL SENSITIVITY ANALYSIS | The platform allow One at time (OAT)but  No global SA | OAT  No OTA  No global SA | No OAT  No global SA | The platform allow One at time (OAT) but  No global SA | No OAT  No global SA |
| PLATFORM/ MODEL VALIDATION, reference | The platform has been largely validated using human biomonitoring data from Europe and the USA, for a variety of compounds covering a large chemical space  Includes metabolism | Done with 15 chemicals (Jongeneelen & ten Berge, 2011 & 2012)  Includes metabolism  Validation needs to be performed on case by case | Not known - test and validate the software tool the project aimed to generate new fit-for-purpose biomarker data from a human volunteer study. | This is clearly described in the model documentation. | Validation needs to be performed on case by case  (does not include metabolites) |
| QSARs as input | YES | YES | YES | YES | YES |
| Single chemicals or mixtures | Single | Single | Not reported. | Single | Single  Consideration of multiple exposure pathways for multiple chemicals to estimate combined exposure of humans and biota |
| Exposure route | oral, dermal, or inhalation routes | oral, dermal, or inhalation routes | oral, dermal, or inhalation routes | Oral, or inhalation routes. No dermal. | Oral and inhalation routes. No dermal. |
| Relevant Data | Default | Database  Imbedded for 15 chemicals info and info for population | Database library | Default | Database library |
| Life-stage simulation  Gender simulation | YES | YES | Not specified | YES | YES |
| Population simulation libraries | Yes  Consumer exposure | YES manual entry | YES | general population, children at different ages, pregnant women  No dermal exposure | YES, but only US population Ring et al., 2017 |
| MODEL USE and OUTPUTS | online and model scenarios and results are stored on the servers of the model developers | Downloadable excel sheet simulation can be saved on model users’ PC | Downloadable excel sheet simulation can be saved on model users’ PC | stores simulations on the model users’ PC | Downloadable - simulation can be saved on model users’ PC |

**S3 Paramaters applied for predictions in IndusChemFate**

**Table S3.1**: Parameter values applied for predictions on DnBP, BBzP, bisphenol A, triclosan and their metabolites with IndusChemFate

| **Abb.** | **Chemical name** | **Metabolite of** | **CAS** | **Molecular Weight (g/mol) (EPA )** | **Density (mg/cm3 or grams/litre)** | **Vapour Pressure (Pa)** | **Log(Kow) in blood pH 7.4** | **Water Solubility (mg/litre)** |
| --- | --- | --- | --- | --- | --- | --- | --- | --- |
| DnBP | Dibutyl phthalate | -/- | 84-74-2 | 278 | 1,050 (av est) | $2.68\times{10}^{-3}$ | 4.50 | 11.2 |
| MnBP | Mono-n-butyl phthalate | DnBP | 131-70-4 | 222 | 1,180 (av est) | $1.88\times{10}^{-3}$  (av est) | 2.77  (av est) | 769  (av est) |
| BBzP | Butylbenzyl phthalate | -/- | 85-68-7 | 312 | 1,130 (av est) | $1.01\times{10}^{-3}$ | 4.73 | 2.69 |
| MBzP | Mono-benzyl phthalate | BBzP | 2528-16-7 | 256 | 1,270 (av est) | $1.26\times{10}^{-4}$  (av est) | 2.80  (av est) | 119  (av est) |
| TCS | Triclosan | -/- | 3380-34-5 | 289 | 1,510 (av est) | $1.30\times{10}^{-3}$  (av est) | 4.76 | 9.99 |
| TCS-glu | Triclosan glucuronide | TCS | 63156-12-7 | 463 | 1,780 (av est) | $3.80\times{10}^{-8}$ (av est) | 2.26  (av est) | 73.7 |
| BPA | Bisphenol A | -/- | 80-05-7 | 228 | 1,170 (av est) | $1.12\times{10}^{-4}$ (av est) | 3.32 | 120 |

Abbreviations; -/- : not applicable; (av est): average estimate; Reference: All parameter values illustrated in this table are sourced
from U.S. EPA, 2019; up to three significant figures are reported.

**Table S3.2:** Parameter values applied for predictions on DnBP, BBzP, bisphenol A, triclosan and their metabolites with IndusChemFate

| **Abb.** | **Vmax Liver (parent[total] μmol/kg tissue/hr)** | **Km Liver (parent[total] μmol/litre)** | **Enterohepatic removal (relative to liver venous blood)** | **Polar Surface Area (PSA) (A^2)** | **Hydrogen Bond Donor (HBD)** | **Oral absorption rate k_a_ (1/h)** | **Elimination half-life (t_1/2_) (h)** | **Time to steady state (estimated; t_1/2_*5) (h)** |
| --- | --- | --- | --- | --- | --- | --- | --- | --- |
| DnBP | 41,280^1^ | 99.7^1^ | 1^2^ | 52.6^3^ | 0^3^ | 9.85^4^ | 6^5^ | 30 |
| MnBP | -/- | -/- | 1^2^ | -/- | -/- | -/- | 2.6^6^ | 13 |
| BBzP | 173,040^7^ | 16.1^7^ | 1^8^ | 52.6^3^ | 0^3^ | 10.7^4^ | 24^9^ | 120 |
| MBzP | -/- | -/- | 1^8^ | -/- | -/- | -/- | 24^9^ | 120 |
| TCS | 34.35^10^ | 122.5^10^ | 0^11,12^ | 29.5^3^ | 1^3^ | 10.8^4^ | 29^11^ | 145 |
| TCS-glu | -/- | -/- | 0^11,12^ | -/- | -/- | -/- | 29^11^ | 145 |
| BPA | 11,304^13^ | 45.8^13^ | 0.2^14,15^ (est) | 40.5^3^ | 2^3^ | 2.8^4^ | 5.4^16^ | 27 |

Abbreviations; -/- : not applicable; (est): estimated; For none of the chemicals selected, information on resorption in renal tubuli was found in the literature. Therefore, for all chemicals a question mark (?) was entered as input value which, according to the documentation in ICF, means that this value is unknown. The time to reach steady state was assessed using the equation T_ss_=5*t_(1/2)_, with t_(1/2)_ being the elimination half-life. Properties of the BPA glucuronide used are those saved in the IndusChemFate model; hence BPA-glu is not included in this table.

References: ^1^Hanioka et al., 2012; ^2^ATSDR, 2018; ^3^U.S. National Center for Biotechnology Information, 2018; ^4^calculated using PSA, HBD and the Winiwarter et al. (1998) model 3b to derive the logarithm of the effective permeability (P_eff_) and Peters (2008, Eq. 1) to then calculate the k_a_; ^5^Aylward et al., 2009; ^6^Koch et al., 2012; ^7^Takahara et al., 2014; ^8^Eigenberg et al., 1986; ^9^European Commission, 2007; ^10^Ashrap et al., 2017; ^11^European Commission Scientific Committee on Consumer Safety (SCCS), 2009; ^12^ECHA, 2015; ^13^Coughlin et al., 2012; ^14^Teeguarden et al., 2005; ^15^Yang et al., 2015; ^16^Völkel et al., 2002.

**S4 Full evaluation of ICF and Httk modelling platforms**

The reliability of a model may be defined as the opposite to its overall uncertainty. The uncertainty of a model addresses both concepts of accuracy and precision. It includes the uncertainty of the input data as well as the sources of uncertainty which are inherent to the model structure (EMA, 2019).

The confidence in the model refers to a judgement by the users of the model – either personal or consensual – on how much they can rely on the model output for certain purposes (EMA, 2019).

The level of confidence in the PBK model platforms used for this exercise can be built based on i. scientific principles; ii. quality of input parameters iii. ability of model to reproduce independent empirical data (WHO, 2010), and can be characterize on the basis of:

1. **The biological basis of the model structure and parameters**

Do the model structure and parameters have a reasonable biological basis?

Are the tissue volumes and tissue blood flow rates within the documented range for the particular species and life stage?

Is the ventilation:perfusion ratio specified in the model within physiological limits?

Is the allometric scaling of parameters, if applicable, done appropriately?

Is the sum total of the tissue blood flow rates equal to the cardiac output?

1. **Comparison of model simulation with experimental data**

How well does the PBPK model reproduce the chemical-specific PK data under various experimental or exposure conditions?

1. **Reliability of model predictions of dose metrics relevant to risk assessment**

How reliable is the PBPK model with regard to its predictions of dose metrics relevant to risk assessment?

**S4.1: Evaluation of IndusChemFate**

IndusChemFate was evaluated using the available chemicals in the library. Bishpenol A was used as chemical to perform the evaluation

Table S4.1: Evaluation of IndusChemFate based on WHO (2010) and OECD (2020) criteria

| **Model evaluation aspect (WHO, 2010 and OECD 2020 CRITERIA)** | **Observations** |
| --- | --- |
| **Biological basis:**  1. Do the model structure and parameters have a reasonable biological basis?  2. Is the sum total of the tissue blood flow rates equal to the cardiac output?  3. Are the tissue volumes and tissue blood flow rates within the documented range for the particular species and life stage?  4. Is the allometric scaling of parameters, if applicable, done appropriately? | 1. YES. The model contains 11 body compartments (Lung, Heart, Brain, Skin, Adipose, Muscles, Bone, Bone marrow, Stomach & Intestines (lumped), Liver and Kidney). The model holds the physiology of human subjects (adult males, adult females, children). Standardized physiology parameters of humans with normal weight and obese are used to dimension organ and tissue volumes and blood flows through these tissues. These parameters are scaled relative to the total body weight.  2. YES  3.YES see manual at page 49  4. Vmax and Km collected from literature were up-scaled to the appropriate unit |
| **Model simulations of data**  5. How well does the PBPK model reproduce the chemical-specific PK data under various experimental or exposure conditions?  6. mass balance is respected?  7. availability of equations and model code | .  5. YES. The model adequately reproduces the chemical specific PK. The model contains published and in-house developed algorithms (QSPRs = Quantitative Structure-Property Relationships) for blood:air partitioning, tissue:blood partitioning and renal excretion  6. The total absorbed mass of the parent compound (“sum inhaled” + ”sum skin air” + “sum skin liquid” + “sum oral absorption” = “total absorbed”) must be equal to the sum of mass of parent + metabolites in all model compartments plus the mass excreted in urine and/or exhaled air (“sum tissues” + “sum air” + “sum blood” + “sum urine” + “sum hep circ” + “sum metabolites lost” = “sum total” ).The total absorbed = the intake dose, however, is slightly higher than the sum of mass, 3% dicrepancy. This could be explained by rounding the numbers in the calculation input and output.  7. YES equations underpinning model structure are available in the manual. Model code available in the Macro under developer. |
| **Reliability (model testing, uncertainty and sensitivity)**  8. How reliable is the PBPK model with regard to its predictions of dose metrics relevant to risk assessment? | 8. the model prediction are reliable (4 to 8 fold) are within the order of magnitude of the HBM data in urines.  A OAT SA was performed to evaluate the impact in changing of 10% of the Vmax and Km and the organ and body weight. Great impact was achieved when changing the Vmax and Km on the urinary excretion concentrations. |

| **Variability/Uncertainty in the Parameter Estimates** | | |  |  |
| --- | --- | --- | --- | --- |
|  | | High | Medium | Low |
| **SENSITIVITY** | High | LogKow |  |  |
|  | Medium |  | Vmax & Km |  |
|  | Low | Variation in human population subjects |  | Body weight and organ weight (variation within the biological limits) |

Figure S4.1.1: Output of the variability/uncertainty and sensitivity analyses for IndusChemFate

**HIGH**

**LOW**

**Model simulations of data; predictivity**

**Biological basis**

There is a 3 to 4 fold overestimation of urinary concentration.

Biological basis are covered and PK are simulated, however, there is a 3% discrepancy of the structure mass balance.

.

**LEVEL OF CONFIDENCE**

**Variability/ Uncertainty in Parameter Analysis; Global Sensitivity Analysis**

A list of assumption and uncertainties is reported and an OAT was performed.

Figure S4.1.2: Illustrative scale of confidence levels of predictions with IndusChemFate

**Overall conclusion for IndusChemFate based on the WHO evaluation scheme with respect to bisphenol A predictions:**

Based on the available information reported and analysis performed using IndusChemFate a standard overall evaluation could be drawn. The IndusChemfate could simulate within the order of magnitude the excreted urinary concentration of BPA. There is a systematic 3% error in the mass balance. To this end the information can be used to support the regulatory assessment but only as informative and/or for prioritization and screening.

**S4.2: Evaluation of R/httk**

Table S4.2: Evaluation of R/httk based on WHO (2010) and OECD (2020) criteria

| **Model evaluation aspect (WHO, 2010 and OECD 2020 CRITERIA)** | **Observations** |
| --- | --- |
| **Biological basis**:  1. Do the model structure and parameters have a reasonable biological basis?  2. Is the sum total of the tissue blood flow rates equal to the cardiac output?  3. Are the tissue volumes and tissue blood flow rates within the documented range for the particular species and life stage?  4. Is the allometric scaling of parameters, if applicable, done appropriately? | 1. YES.The model contains 7 body compartments. The model holds libraries of population reporting the physiology of human subjects (adult males, adult females, children). Parameters are scaled relative to the total body weight. Overall, CL_tot_ is biased toward underestimation; parameters characterising CL_tot_ are hepatocellular clearance and plasma protein binding. Tissue-specific partition coefficients (except red blood cells) were calibrated through comparison to *in vivo* measured partition coefficients.  2. YES  3. YES  4. Hepatocellular clearance and fraction unbound values when needed are scaled up to total organ weight. |
| **Model simulations of data**  5. How well does the PBPK model reproduce the chemical-specific PK data under various experimental or exposure conditions?  6. mass balance is respected?  7. availability of equations and model code | 5. YES. The model adequately reproduces the chemical specific PK.  The model QSAR algorithms for partitioning between tissues and blood, for uptake and renal excretion.  6. YES  7. YES equations underpinning model structure are available in the manual. Model code available in R. |
| **Reliability (model testing, uncertainty and sensitivity)**  8. How reliable is the PBPK model with regard to its predictions of dose metrics relevant to risk assessment? | 8. the model prediction are reliable (4 to 8 fold) are within the order of magnitude of the HBM data in urines. |

| **Variability/Uncertainty in the Parameter Estimates** | | |  |  |
| --- | --- | --- | --- | --- |
|  | | High | Medium | Low |
| **SENSITIVITY** | High |  | CL_tot_ (renal clearance, metabolic clearance) | Tissue-specific partition coefficients |
|  | Medium | V_d_ (for BPA)  F_bio_ (for BPA and TCS) |  | Physico-chemical properties, f_up_, |
|  | Low |  |  | Body weight and organ weight (variation within the biological limits)  Variation in human population subjects |

Figure S4.2.1: Output of the variability/uncertainty and sensitivity analyses for R/httk; V_d_ = volume of distribution; F_bio_ = bioavailability; f_up_ = fraction unbound in plasma

Figure S4.2.2: Illustrative scale of confidence levels of predictions with R/httk

**HIGH**

**LOW**

**Model simulations of data; predictivity**

**Biological basis**

Model predictions are reliable (4 to 8 fold), within the order of magnitude

Biological basis are covered and PK are simulated, however, metabolism was not available

.

**LEVEL OF CONFIDENCE**

**Variability/ Uncertainty in Parameter Analysis; Global Sensitivity Analysis**

A list of assumption and uncertainties is reported and an OAT was performed.

**Overall conclusion for R/httk based on the WHO evaluation scheme with respect to bisphenol A and triclosan predictions:**

Based on the available information reported and analysis performed using R/httk a standard overall evaluation could be drawn. The R/httk could simulate within the order of magnitude the excreted urinary concentrations. The model runs without errors but does not include metabolism. To this end the information can be used to support the regulatory assessment but only as informative and/or for prioritization and screening.

**S5 Additional information and results of simulations**

**Table S5.1:** Times to steady state and selected simulation time for each compound run in Httk

|  | **Calc_css results (days)** | **Simulation time (days)** |
| --- | --- | --- |
| **MeP** | 1 | 7 |
| **EtP** | 1 | 7 |
| **PrP** | 2 | 7 |
| **BuP** | 2 | 7 |
| **TCS** | 4 | 10 |
| **BP-3** | 2 | 7 |
| **BPA** | 11 | 30 |

**Table S5.2**: Estimated Daily Intake (EDI) values for Danish children aged 6-10 years (taken from Frederiksen et al., 2013).

|  | boys | | girls | |
| --- | --- | --- | --- | --- |
| mg/kg BW/day | 50th percentile | 95th percentile | 50th percentile | 95th percentile |
| BPA | 0.0000797 | 0.000313 | 0.0000497 | 0.000316 |
| TCS | 0.0000278 | 0.013761 | 0.0000188 | 0.012345 |

**Table S5.3**. Comparison of urinary concentrations of two Norwegian female adults (1-normal weight, 2-obese) to ICF simulated BE_EDI_ (EDI-based urine concentrations) and predicted BE_RfD_ and BE_TDI_ values after 24 hours. EDI values were estimated based on concentrations measured in external sources (food, air, dust).

|  | **Woman** | **Parent compound** | **Metabolite** | **TDI (mg/kg/** | **RfD (mg/kg/** | **BE_TDI_ (ng/mL)** | **BE_RfD_ (ng/mL)** | **Factor measured urine conc. / BE_EDI_** | **Exceedance of threshold (conc. In urine/BE_TDI_)** | **Exceedance of threshold (conc. In urine/BE_RfD_)** |
| --- | --- | --- | --- | --- | --- | --- | --- | --- | --- | --- |
|  |  |  |  | **day)** | **day)** |  |  |  |  |  |
| Phthalates | 1 | DnBP | MnBP | 0.01 | 0.1 | 0.14 | 1.37 | 8875.0 | **111.57** | **11.40** |
|  | 2 | DnBP | MnBP | 0.01 | 0.1 | 0.13 | 1.34 | 18508.3 | **128.85** | **12.50** |
|  | 1 | BBzP | MBzP | 0.5 | 0.2 | 7.8 | 3.12 | 7769.5 | 0.67 | **1.66** |
|  | 2 | BBzP | MBzP | 0.5 | 0.2 | 7.66 | 3.06 | 11371.0 | 0.37 | 0.92 |
| Phenols | 1 | TCS | TCS-glu | 0.047 | 0.3 | 0.44 | 2.67 | - | 0.16 | 0.04 |
|  | 2 | TCS | TCS-glu | 0.047 | 0.3 | 0.39 | 2.36 | - | 0.12 | 0.03 |
|  | 1 | BPA | BPA-glu | 0.004 | 0.05 | 377 | 4709 | 0.1 | 0.0050 | 0.0004 |
|  | 2 | BPA | BPA-glu | 0.004 | 0.05 | 437 | 5462 | 9.3 | 0.0251 | 0.0020 |

**Table S5.4**. Comparison of urine concentrations of two Norwegian children to ICF simulated BE_EDI_ and predicted BE_RfD_ and BE_TDI_ values after 24 hours. EDI values were estimated based on concentrations measured in external sources (food, air, dust).

|  | Child | Parent compound | Metabolite | TDI (mg/kg/ | RfD (mg/kg/ | BE_TDI_ (ng/mL) | BE_RfD_ (ng/mL) | Factor measured urine conc. / BE_EDI_ | Exceedance of threshold (conc. In urine/BE_TDI_) | Exceedance of threshold (conc. In urine/BE_RfD_) |
| --- | --- | --- | --- | --- | --- | --- | --- | --- | --- | --- |
|  |  |  |  | **day)** | **day)** |  |  |  |  |  |
| Phthalates | 1 | DnBP | MnBP | 0.01 | 0.1 | 0.23 | 2.26 | 1511.71 | **72.96** | **7.42** |
|  | 2 | DnBP | MnBP | 0.01 | 0.1 | 0.23 | 2.26 | 17553.19 | **251.09** | **25.55** |
|  | 1 | BBzP | MBzP | 0.5 | 0.2 | 10.89 | 4.36 | 1215.25 | 0.25 | 0.62 |
|  | 2 | BBzP | MBzP | 0.5 | 0.2 | 10.9 | 4.36 | 10554.37 | 0.91 | 2.27 |
| Phenols | 1 | TCS | TCS-glu | 0.047 | 0.3 | 0.73 | 4.46 | - | 0.08 | 0.02 |
|  | 2 | TCS | TCS-glu | 0.047 | 0.3 | 0.67 | 4.11 | - | 0.05 | 0.01 |
|  | 1 | BPA | BPA-glu | 0.004 | 0.05 | 637 | 7968 | 16.17 | 0.0166 | 0.0013 |
|  | 2 | BPA | BPA-glu | 0.004 | 0.05 | 736 | 9194 | 0.56 | 0.0020 | 0.0002 |

**Table S5.5**. Comparison of urine concentrations of four individuals to ICF simulated BE_EDI_ and the predicted BE_RfD_ and BE_TDI_ of BPA at steady state. EDI values were estimated based on concentrations measured in external sources (food, air, dust).

| Woman | Parent compound | Metabolite | TDI (mg/kg/  day) | RfD (mg/kg  /day) | BE_TDI_ (ng/mL) | BE_RfD_ (ng/mL) | Factor measured urine conc. / BE_EDI_ | Exceedance of threshold (conc. In urine/BE_TDI_) | Exceedance of threshold (conc. In urine/BE_RfD_) |
| --- | --- | --- | --- | --- | --- | --- | --- | --- | --- |
| 1 | BPA | BPA-glu | 0.004 | 0.05 | 427.92 | 5348.90 | 0.1257 | **0.0044** | **0.0004** |
| 2 | BPA | BPA-glu | 0.004 | 0.05 | 473.09 | 5913.60 | 8.5932 | **0.0232** | **0.0019** |
| Child | Parent compound | Metabolite | TDI (mg/kg/day) | RfD (mg/kg/  day) | BE_TDI_ (ng/mL) | BE_RfD_ (ng/mL) | **Factor measured urine conc. / BE EDI** | **Exceedance of threshold (conc. In urine/BE_TDI_)** | **Exceedance of threshold (conc. In urine/BE_RfD_)** |
| 1 | BPA | BPA-glu | 0.004 | 0.05 | 649.20 | 8114.88 | 0.0630 | **0.0163** | **0.0013** |
| 2 | BPA | BPA-glu | 0.004 | 0.05 | 739.19 | 9239.74 | 1.7986 | **0.0020** | **0.0002** |

**Figure S5.1**. Hazard Index calculated with the 95^th^ percentile concentration of a chemical in urine with respect to the BE value established by selecting the 5^th^ percentile.

**Figure S5.2**. Hazard Index calculated with the median concentration of a chemical in urine with respect to the BE value established by selecting the 5^th^ percentile.

**References**

Ashrap, P. *et al.* (2017) ‘Discovery of a widespread metabolic pathway within and among phenolic xenobiotics’, *Proceedings of the National Academy of Sciences of the United States of America*, 114(23), pp. 6062–6067. doi: 10.1073/pnas.1700558114.

ATSDR (2018) Agency for Toxic Substances and Disease Registry. Available at: https://www.atsdr.cdc.gov (Accessed: 16 August 2018).

Aylward, L. L. *et al.* (2009) ‘Derivation of Biomonitoring Equivalents for di-n-butyl phthalate (DBP), benzylbutyl phthalate (BzBP), and diethyl phthalate (DEP)’, *Regulatory Toxicology and Pharmacology*, 55(3), pp. 259–267. doi: 10.1016/j.yrtph.2009.09.003.

Boas M, Frederiksen H, Feldt-Rasmussen U, Skakkebaek NE, Hegedus L, Hilsted L, Juul A & Main KM 2010 Childhood exposure to phthalates: associations with thyroid function, insulin-like growth factor I, and growth. Environmental Health Perspectives 118 1458–1464. (doi:10.1289/ehp.0901331)

Boeniger, M. F., Lowry, L. K. and Rosenberg, J. (1993) ‘Interpretation of Urine Results Used to Assess Chemical Exposure with Emphasis on Creatinine Adjustments: A Review’, American Industrial Hygiene Association Journal, 54(10), pp. 615–627. doi: 10.1080/15298669391355134

Cequier, E. et al. (2015) ‘Human exposure pathways to organophosphate triesters - A biomonitoring study of mother-child pairs’, Environment International. Elsevier Ltd, 75, pp. 159–165. doi: 10.1016/j.envint.2014.11.009

Cequier, E. et al. (2017) ‘Exposure to organophosphorus pesticides in Norwegian mothers and their children: Diurnal variability in concentrations of their biomarkers and associations with food consumption’, Science of the Total Environment. Elsevier B.V., 590–591, pp. 655–662. doi: 10.1016/j.scitotenv.2017.03.017.

Coughlin, J. L., Thomas, P. E. and Buckley, B. (2012) ‘Inhibition of genistein glucuronidation by bisphenol A in human and rat liver microsomes’, *Drug Metabolism and Disposition*, 40(3), pp. 481–485. doi: 10.1124/dmd.111.042366.

ECHA (2015) Regulation (EU) No 528/2012 concerning the making available on the market and use of biocidal products. Evaluation of active substances. Assessment Report Triclosan. Product-type 1 (Human hygiene). Available at: <http://dissemination.echa.europa.eu/Biocides/ActiveSubstances/1406-01/1406-01_Assessment_Report.pdf>.

EMA (2019). Guideline on the qualification and reporting of physiologically based pharmacokinetic (PBPK) modelling and simulation. European Medicines Agency https://www.ema.europa.eu/en/documents/scientific-guideline/guideline-reporting-physiologically-based-pharmacokinetic-pbpk-modelling-simulation_en.pdf

Eigenberg, D. A. *et al.* (1986) ‘Distribution, excretion, and metabolism of butylbenzyl phthalate in the rat’, *Journal of Toxicology and Environmental Health*, 17(4), pp. 445–456. doi: 10.1080/15287398609530839.

European Commission (2007) European Union Risk Assessment Report, CAS: 85-68-7, benzyl butyl phthalate (BBP). Available at: <https://echa.europa.eu/documents/10162/bad5c928-93a5-4592-a4f6-e02c5e89c299>.

European Commission Scientific Committee on Consumer Safety (SCCS) (2009) Opinion on Triclosan, COLIPA n° P32’. Available at: <https://ec.europa.eu/health/scientific_committees/consumer_safety/docs/sccs_o_054.pdf>.

Hanioka, N. *et al.* (2012) ‘Hydrolysis of di-n-butyl phthalate, butylbenzyl phthalate and di(2-ethylhexyl) phthalate in human liver microsomes’, *Chemosphere*, 89(9), pp. 1112–1117. doi: 10.1016/J.CHEMOSPHERE.2012.05.095.

Koch, H. M. *et al.* (2012) ‘Di-n-butyl phthalate (DnBP) and diisobutyl phthalate (DiBP) metabolism in a human volunteer after single oral doses’, *Archives of Toxicology*, 86(12), pp. 1829–1839. doi: 10.1007/s00204-012-0908-1.

OECD (2020) OECD guidance document on the characterisation, validation and reporting of PBK models for regulatory purposes ((under endorsement)

Pearson, M. a, Lu, C., Schmotzer, B. J., Waller, L. a, & Riederer, A. M. (2009). Evaluation of physiological measures for correcting variation in urinary output: Implications for assessing environmental chemical exposure in children. Journal of Exposure Science & Environmental Epidemiology, 19(3), 336–42. <http://doi.org/10.1038/jes.2008.48>

Peters, S. A. (2008) ‘Evaluation of a generic physiologically based pharmacokinetic model for lineshape analysis’, *Clinical Pharmacokinetics*, 47(4), pp. 261–275. doi: 10.2165/00003088-200847040-00004.

Sakhi, et al 2017, Phthalate metabolites in Norwegian mothers and children: Levels, diurnal variation and use of personal care products, Science of The Total Environment, Volumes 599–600, 2017, Pages 1984-1992, <https://doi.org/10.1016/j.scitotenv.2017.05.109>

Sakhi, et al 2018, Levels, variability and determinants of environmental phenols in pairs of Norwegian mothers and children, Environment International, Volume 114, 2018, Pages 242-251, <https://doi.org/10.1016/j.envint.2018.02.037>

Takahara, Yuka *et al.* (2014) *Butylbenzyl Phthalate Hydrolysis in Liver Microsomes of Humans, Monkeys, Dogs, Rats and Mice*, *Biological and Pharmaceutical Bulletin*. Available at: https://www.jstage.jst.go.jp/article/bpb/37/4/37_b14-00002/_pdf/-char/ja.

Teeguarden, J. G. *et al.* (2005) ‘Evaluation of oral and intravenous route pharmacokinetics, plasma protein binding, and uterine tissue dose metrics of bisphenol A: A physiologically based pharmacokinetic approach’, *Toxicological Sciences*, 85(2), pp. 823–838. doi: 10.1093/toxsci/kfi135.

U.S. National Center for Biotechnology Information (2018) *PubChem*. U.S. National Library of Medicine. Available at: https://pubchem.ncbi.nlm.nih.gov/search/ (Accessed: 9 July 2018).

Völkel, W. *et al.* (2002) ‘Metabolism and kinetics of bisphenol a in humans at low doses following oral administration’, *Chemical Research in Toxicology*, 15(10), pp. 1281–1287. doi: 10.1021/tx025548t.

WHO, (2010). Characterization and application of physiologically based pharmacokinetic models in risk assessment. International Programme on Chemical Safety. Harmonization Project Document No. 9. World Health Organization Available at: <http://www.inchem.org/documents/harmproj/harmproj/harmproj9.pdf>.

Winiwarter, S. *et al.* (1998) ‘Correlation of human jejunal permeability (in vivo) of drugs with experimentally and theoretically derived parameters. A multivariate data analysis approach’, *Journal of Medicinal Chemistry*, 41(25), pp. 4939–4949. doi: 10.1021/jm9810102.

Yang, X. *et al.* (2015) ‘Development of a physiologically based pharmacokinetic model for assessment of human exposure to bisphenol A’, *Toxicology and Applied Pharmacology*, 289, pp. 442–456. doi: 10.1016/j.taap.2015.10.016.
